# Acknowledgment to the Reviewers of *Antioxidants* in 2022

**DOI:** 10.3390/antiox12020230

**Published:** 2023-01-19

**Authors:** Antioxidants Editorial Office

**Affiliations:** MDPI AG, St. Alban-Anlage 66, 4052 Basel, Switzerland

High-quality academic publishing is built on rigorous peer review. *Antioxidants* was able to uphold its high standards for published papers due to the outstanding efforts of our reviewers. Thanks to the efforts of our reviewers in 2022, the median time to first decision was 16 days and the median time to publication was 35 days. Regardless of whether the articles they examined were ultimately published, the editors would like to express their appreciation and thank the following reviewers for the time and dedication that they have shown *Antioxidants*:
Abdelhabib SemlaliKatrina MirandaAbdelouahed KhalilKatsumasa GotoAbdolreza HosseindoustKatsura TakanoAbdul Rashid AzizKatsutaro MorinoAbdullah Md. SheikhKatsutoshi HoriAbe HiroyaKatsuya HirasakaAbel Bult-ItoKatsuyoshi MatsunamiAbhirup DasKaustuv BasuAbraham Wall MedranoKavindra Kumar KesariAdam BenhamKavita SharmaAdam KlimowiczKavitha MenonAdam MatkowskiKay D. BeharryAdel PezeshkiKay-Dietrich WagnerAdele PapettiKazuhiko KotaniAdesh SainiKazuhiro P. IzawaAdil El MidaouiKazuo KobayashiAdina Ionuta GavrilaKazuo N. WatanabeAditi JainKazuo TakahashiAdolfo Andrade CettoKazuomi SatoAdolfo Gabriele MauroKazuya MatsuoAdrià ArboixKazuya ToriumiAdrian FifereKazuyuki KasaharaAdrian HegemanKedar N. PrasadAdrián MatencioKeiichi HironoAdrian ȘpacKeiichi IshiharaAdriana AdameovaKeiko HosohataAdriana Erica MieleKeisuke IshizawaAdriana MikaKeith MartinAdriana SmarandacheKeith MorrisAdriana VallesiKeith RochfortAdrianne BendichKelly S. SantangeloAdriano Costa De CamargoKen ItohAdriano MollicaKenichi GotoAdrie VerhoevenKen-Ichiro MinatoAdrien GeorgesKenji YamadaAenim PaeKenneth AdlerAera JangKenneth OlsonAftab ShaukatKenneth SöderhällAgata CampisiKenneth W. McmillinAgata GórskaKesen MaAgata GóźdźKeshav Raj PaudelAgata ZmijewskaKevin ChenÁgnes CséplőKevin TidgewellÁgnes FarkasKhawar JabranAgnes SchröderKhiena Z. BraininaAgnes SzepesiKhosrow KashfiAgnese De MarioKhurshid AhmadAgnieszka BartoszekKhushwant Singh BhullarAgnieszka Bronowicka-SzydełkoKi Choon ChoiAgnieszka GalantyKieu The Loan TrinhAgnieszka GniazdowskaKijin KimAgnieszka KitaKikuko AmagaseAgnieszka KrupaKilmer S. MccullyAgnieszka KwiatekKim OhlAgnieszka LudwiczukKim StoteAgnieszka Mazur-BialyKimmo RumpunenAgnieszka MicekKinga GawelAgnieszka Olejnik-SchmidtKinga SałatAgnieszka OrkuszKirill V. ZaitsevAgnieszka Owczarczyk-SaczonekKirtikar ShuklaAgnieszka PartykaKit Leong CheongAgnieszka PilarskaKitaoka YuAgnieszka PłażekKi-Yeon YooAgnieszka ŚcibiorKiyoshi AsadaAgnieszka SzewczykKiyoshi FukuharaAgnieszka Szlagatys-SidorkiewiczKlára KosováAgnieszka SzopaKlaus Peter LattéAgnieszka ViapianaKlaus Schmidt-RohrAgnieszka WnukKlaus-Dieter SchlüterAgnieszka Zembron-LacnyKo OkumuraAgostino CasapulloKoga YasuhikoAgustín G. AsueroKogiku ShibaAgustín Llopis-GonzálezKoichi FujisawaAhmad SaltiKoichi HashimotoAhmed BakillahKoji MiyamotoAhmed E. Abdel MoneimKoji NaruishiAhmed El GhorabKomal RamaniAhmed GadKomuraiah MyakalaAhmed HasanKonrad SzychowskiAhmed NoreldinKonstantin ChekanovAhmed SayedKonstantin V. KudryavtsevAikaterini GatsiouKonstantin VolchoAi-Min WuKonstantinos FeidantsisAiming YuKosmas NathanailidesAinhoa Plaza-ZabalaKostantin ChegaevAinhoa RuizKosuke NakamuraAirton C. MartinsKosuke NishiAjaikumar B. KunnumakkaraKrishna Chaitanya SuddalaAjay SharmaKrista MorleyAjit GhoshKristaps KlavinsAkihiko HiyamaKristen RakAkiko Kojima-YuasaKristian HaanesAkiko TanakaKristina LoßowAkimitsu MiyajiKristina LožienėAkira NakajimaKristine GriffettAkira ObanaKristl JanjaAkira SugawaraKristy WelshhansAkiyoshi TakamiKrisztina Takacs-NovakAkriti SrivastavaKrystyna GołembiowskaAlaeldein M. AbudabosKrystyna Pierzchała-KoziecAlain ChapelKrzesimir CiuraAlbena T. Dinkova-KostovaKrzysztof CzajaAlbert MoralesKrzysztof J. FilipiakAlberto BaldelliKrzysztof PolewskiAlberto GallegosKrzysztof SitkoAlberto GonzálezKrzysztof SztankeAlberto Horcada IbáñezKrzysztof SzyfterAlberto MantovaniKrzysztof ZborowskiAlberto OrtizKsenija DurgoAlberto Rodríguez-ArchillaKuan Hung LinAlbino CarrizzoKuan-Chen ChengAlbino MaggioKuang-Tai KuoAlbrecht PiiperKuboyama TomoharuAldo TavaKuiwu WangAlec SimpsonKumar GanesanAlejandra Garcia-AlonsoKung-Woo NamAlejandro CarazoKunio YuiAlejandro Islas-JácomeKunjan R. DaveAlejandro PlascenciaKunka Mohanram RamkumarAlejandro RomeroKun-Lin TsaiAlejandro Samhan-AriasKuo DuAleksandar Ž. KostićKurt FagerstedtAleksandr G. BulaevKwang Yeon HwangAleksandra Duda-ChodakKwang Youl LeeAleksandra GlogowskaKyle TimmermanAleksandra KotyniaKyoung Soo KimAleksandra PawlakKyoung-Chan ParkAleksandra Piechota-PolanczykKyung-Min KimAleksandra S. KristoKyung-Mok ParkAleksandra Wisłowska-StanekKyung-Sun HeoAleksei V. TrofimovLadislav Eav KokoškaAleksei Ya TikhonovLaima ČesonienėAlena PechovaLajos NagyAlena Ribeiro Alves Peixoto MedradoLakshmaiah SreeramaAleš GregorcLana McclementsAlessandra BosuttiLara BarazzuolAlessandra FerramoscaLara CostantiniAlessandra FraternaleLara GibelliniAlessandra GamberucciLara Saftić MartinovićAlessandra GhigoLara Valiño-RivasAlessandra MarescaLarbi RhaziAlessandra MiceraLarisa RyskalinAlessandra PalladiniLars GilleAlessandra RussoLars-Olof BjörnAlessandra StacchiottiLassaâd BelbahriAlessandra VezzoliLászló HazaiAlessandro AlivertiLászló KőrösiAlessandro Di CerboLaszlo TretterAlessandro FratiLászló VécseiAlessandro MarianiLaszlo ViragAlessandro MarroniLaura Alejandra De La RosaAlessia CatalanoLaura BertiniAlessia RemiganteLaura BravoAlessia RosiLaura CiapponiAlessio AdamianoLaura CorteseAlessio BocediLaura FavettaAlessio ZulianiLaura G. SherlockAletras AlexiosLaura GambariAlex J. B. KreutzbergerLaura Giuseppina Di PasquaAlex MichelsLaura GrasaAlexander AngerhoferLaura IeloAlexander BarteltLaura LocatelliAlexander BaykovLaura LombaAlexander BerezinLaura Lõpez-PingarrõnAlexander BetekhtinLaura OrianAlexander K. GoroncyLaura RizziAlexander KarichLaura SantagostiniAlexander O. ChizhovLaura SiracusaAlexander S. LukatkinLaureano De La VegaAlexander Valer’Evich DushkinLauren ErlandAlexandra FidalgoLaurence LeherteAlexandra Ripszky TotanLaurence ParnellAlexandros GeorgakilasLayla Al-NakkashAlexey F. TopunovLea D. BennettAlexey V. FedulovLea SpindlerAlexey V. FeofanovLeandro Licursi De OliveiraAlexios Vlamis-GardikasLecturer Carmen Rodica PopAlexis GalanisLee RichardAlfonsina GattusoLei WangAlfonso Olaya AbrilLeif BulowAlfonso SchiaviLeilson BezerraAlfonso Varela-LópezLenka CeslovaAlfonso ZambonLenka VorlováAlfredo CaturanoLenuta-Maria SutaAlfredo GrilliLeonardo AugustoAlfredo Saavedra-MolinaLeonel E. RojoAlfredo TeixeiraLeonel PereiraAli KoskelaLeonid V. KulikAlica PizentLeopold EckhartAlice BonomiLeroy C. JosephAlice S. PereiraLeslie MyattAlicia Rodríguez-BarberoLeszek KubiszAlicja KluczykLetizia Li PianiAlin CiobicaLeto-Aikaterini TzivelekaAlina BoraLeyland FraserAlina Kunicka-StyczyńskaLi XiaoAlina Pyka-PająkLia NakaoAlison GrayLia RimondiniAlkmini T. AnastasiadiLiana SilvaAlla IvanovaLi-Ang LeeAllen HerbstLiangcan HeAlp SenerLiangfa GeAltaf MohammedLiang-Yu ChenAlvise SernicolaLibor KvitekAlyssa Hidalgo VidalLibor VitekAmala SoumyanathLídia GonçalvesAmanda GendersLigen LinAmandine CaruanaLi-Han HsuAmany TawfikLi-Jen LeeAma-Tawiah EssilfieLilian Calderon-GarciduenasAmelia BarilliLiliana CepoiAmitava MukherjeeLiliana Mititelu TartauAmro AbulilaLiliya S. LogoydaAna AlcudiaLin WangAna ArnaizLin ZhaoAna B. CerezoLina CossignaniAna Belén Díaz SánchezLina RaudonėAna Belén Sabater-JaraLinda PetersAna Bucić-KojićLindsey KennedyAna BugaLing LiAna Čipak GašparovićLingwen DingAna Clara CristóvãoLingxia ZhaoAna Cristina Agulheiro SantosLisa A. DelouiseAna FernandesLisa DeanAna FloresLisa GiovannelliAna Guerra-LibreroLisardo BoscaAna I. ArrobaLisete PaivaAna I. ReyLiubov N. SkrypnikAna Isabel EsquifinoLivius V. D’UscioAna Isabel FernandezLong ChenAna Isabel PrietoLongshuang HuangAna José Jiménez-AraujoLoredana DumitrascuAna Jurinjak TusekLoredana SalernoAna Laura De La GarzaLorelei Irina BrasoveanuAna LedoLorena Martínez-ZamoraAna LimaLorena UrbanelliAna Lúcia FerreiraLorenz S. NeuwirthAna Luísa De Sousa-CoelhoLorenza TrabalziniAna M. TroncosoLorenzo CecchiAna Margarida SilvaLorenzo MantiAna María García-BoresLoretta LazzaratoAna María GutiérrezLoris PintoAna María Sánchez-PérezLouis IrvingAna MaroteLouis Kuoping ChaoAna MornarLouis PenningAna MuñozLouise E. See HoeAna ReisLourdes M. VarelaAna SayagoLowell D. KispertAna SeguraLu HanAna StupinLu WangAna Teresa SerraĽubomíra TóthováAna Varela CoelhoLuc NegroniAnacharis B. Sá-NakanishiLuca CalaniAna-Maria Chiorcea-PaquimLuca CuculloAnandh Babu Pon VelayuthamLuca SettanniAnanth PannalaLuca ValgimigliAnastasia KotanidouLuca VanellaAnastasia TsingotjidouLucas R. SmithAnastasia ZervaLucia A. SealeAnastasiia KrivoruchkoLucia ChiummientoAnbazhagan SathiyaseelanLucia GrengaAnca DinischiotuLucia MarcocciAnca DumbravaLucia MorbidelliAnca GafencuLucia PanzellaAnca Lucau-DanilaLucian HriţcuAnca PanaitescuLuciana AzevedoAnca ZanfirescuLuciana BordinAndrás BikovLuciana CorrêaAndrás MihályLuciana HannibalAndras PerlLuciana MandrioliAndrás SzarkaLuciano PinottiAndre AraujoLucie BrissonAndré L. SimãoLucjusz ZaprutkoAndre LechelLucyna MrówczyńskaAndré Rolim BabyLudek SlavikAndrea AngelettiLudmila KazdovaAndrea AntonelliLudmila V. PuchkovaAndrea ArsiccioLudovico AbenavoliAndrea BaschieriLuigi DonatoAndrea BuneaLuigi LuciniAndrea FinLuigi MenghiniAndrea GalliLuigi MilellaAndrea Herrero-CerveraLuigi RosatiAndrea HricováLuigi SansoneAndrea PautzLuigi SantacroceAndrea Pereira RegnerLuis Alberto Cira ChávezAndrea PerrelliLuis AlvesAndrea RagusaLuis Angel Medina JuarezAndrea RomaniLuis Ángel Rubio San MillanAndrea SansoneLuis David Solis MurgasAndrea TarozziLuis GoyaAndrea TrevisanLuis M. MateosAndrea VasconsueloLuis Noguera ArtiagaAndrea VenerandoLuís PatarataAndreas EisenreichLuís PintoAndreas K. NüsslerLuísa CarvalhoAndreas KoschellaLuisa Di PaolaAndreas PetryLukáš KubalaAndreas SimmŁukasz BobakAndreas Von KnethenLukasz MalekAndreea StanilaŁukasz SkoczylasAndrei I. KhlebnikovŁukasz SobottaAndreia N. CarvalhoŁukasz ŚwiątekAndreja R. LeskovacŁukasz SzeleszczukAndres Alfredo Pech-CervantesŁukasz WojtylaAndrés MorenoLuke SimonAndrew D. RedfernLuminita DavidAndrew DancisLuna Maslov BandićAndrew DinardoLusânia AntunesAndrew Ian JoblingLu-Sheng HsiehAndrew QuestLydia Giménez-LlortAndrey A. ZamyatninM. Azizur RahmanAndrey AbramovMª Carmen Duran-RuizAndrey DrozdovMaaike KockxAndrey Zamyatnin, Jr.Macarena Celina Cáceres LeónAndriana C. KalioraMacdonald WickAndromachi TzaniMaciej JarzebskiAndrzej BajguzMaciej OziembłowskiAndrzej DawidowiczMaciej StrzemskiAndrzej GrzybowskiMaciej TarnowskiAndrzej PółtorakMaciej WnukAndrzej SlominskiMadhavan NampoothiriAndrzej SzutowiczMadhavi Latha GandlaAndy Wai Kan YeungMadhumita ChatterjeeAnelia G. DobrikovaMagda PálAnestis KarkanisMagdalena Chottova DvorakovaAneta BalcerczykMagdalena Jeszka-SkowronAneta KopećMagdalena KrauzeAneta StachowiczMagdalena Lebiedzinska-ArciszewskaAneta WojdyłoMagdalena Martínez-CañameroAng LiMagdalena Martínez-ToméAngel Gil-IzquierdoMagdalena MititeluÁngel Isidro Cámpa-CórdovaMagdalena Orczyk-PawiłowiczAngel L. OrtegaMagdalena PaczkowskaAngel L. PeyMagdalena RudzińskaAngel Luis García-VillalónMagdalena RyśAngela BisioMagdalena StobieckaAngela BriegerMagdalena Wirkowska-WojdyłaAngela De SimoneMagdalena WójciakAngela FaddaMagdalene J. SeilerAngela MessinaMaha CouchaÁngeles DomínguezMahendra D. ChrodiaAngélica María Sabogal-GuáquetaMahendra SinghAngelika MustrophMaher Y. AbdallaAngelo D’AlessandroMahmoud KandeelAngelo GismondiMaja BenkovićAngelo SpadaroMaja Jazvinšćak JembrekAnibal De Freitas Santos JuniorMaja MatulicAniello FrancescoMaja PotokarAnika WagnerMakio TakedaAniko GorbeMakoto MakishimaAnil SinghMakoto TsunodaAnita BiesiadaMakusu TsutsuiAnita PichlerMałgorzata BiałekAnitra CarrMalgorzata Czystowska-KuzmiczAnja JoachimMałgorzata DudaAnkit RochaniMałgorzata GniewoszAnkita BurmanMałgorzata GrabarczykAnkush PrasadMałgorzata GrembeckaAnn B. MoserMałgorzata IciekAnna ArnoldiMałgorzata JeleńAnna AtlanteMałgorzata Kotula-BalakAnna BaldisserottoMalgorzata KucinskaAnna BelcarzMałgorzata KujawskaAnna Bilska-WilkoszMałgorzata KwiecieńAnna BizońMalgorzata Latos-BrozioAnna BrancatoMałgorzata Lewandowska-SzumiełAnna Cleta CroceMałgorzata MaterskaAnna CzarneckaMałgorzata Moczkowska-WyrwiszAnna CzechMalgorzata NowackaAnna D. SerefkoMałgorzata OlszowyAnna Di SalleMałgorzata PrzybyłoAnna ErmundMalgorzata RozanowskaAnna FreemanMałgorzata StarowiczAnna GalickaMałgorzata ŚwiątkiewiczAnna Gramza-MichałowskaMałgorzata SzczukoAnna Herman-AntosiewiczMałgorzata TabaszewskaAnna JakubczykMalgorzata Tyszka-CzocharaAnna Julie PeiredMałgorzata WojciechowskaAnna Kablak-ZiembickaMałgorzata WojtkowskaAnna Kostecka-GugalaMałgorzata WrzosekAnna KulikMalgorzata Zakłos-SzydaAnna LewinskaMalgorzata ZawadzkaAnna M. MastrangeloMaliha ZahidAnna Maria BassiMamoru IsemuraAnna Maria SardanelliMamoru TakedaAnna MertasManish Vijaya KumarAnna Merwid-LądManmeet BhallaAnna Michalak-StomaMann-Jen HourAnna OlejnikManon CoutureAnna OniszczukMansour SobehAnna PannaccioneManuel BlázquezAnna PetruczynikManuel De-MiguelAnna R. MalikManuel Espinosa UrgelAnna Rita BiliaManuel GarrosaAnna Rzepecka-StojkoManuel Marques FerreiraAnna SansoneManuel Moya-VilarAnna SerefkoManuel RenduelesAnna SignorileManuel Viuda-MartosAnna StefanskaManuela LeriAnna StojakowskaManuela StefanelliAnna Tabęcka-ŁonczyńskaManuela Vaz-VelhoAnna TrusekManuela ViolaAnna ValentinoMar Fernandez GutierrezAnna VaskuMarc FransenAnna Vittoria MattioliMarc HerbAnna Winiarska-MieczanMarc YesteAnna ŻbikowskaMarc-André LécuyerAnnabel S. BerthonMarcel BonayAnnagrazia AdornettoMarcel KrzanAnnalisa ChiavaroliMarcel PârvuAnnalisa CurcioMarcel ZamockyAnnalisa MaiettiMarcello CasertanoAnnalisa NoceMarcello CeciAnnalisa SantucciMarcelo FerreiraAnnarita FetoniMarcelo FranchinAnnarita PanighelMarcelo Larami SantoroAnne BlaisMarcelo N. MuscaraAnne Dragøy HafstadMarcelo Sousa SilvaAnne J. AndersonMarcin ArciszewskiAnne JarryMarcin BarszczAnne JörnsMarcin CichoszAnne VirsolvyMarcin GnybaAnnelie DamerauMarcin ŁukasiewiczAnne-Louise PonsonbyMarcin OżarowskiAnnette GrahamMarcin SamiecAnnunziata NuscaMarcin SzymańskiAnroop B. NairMarcin ZielińskiAnthony HaighMárcio José De Abreu RodriguesAnthony J. BaucumMárcio RodriguesAntien L. MooyaartMarco AlbanoAnton AirineiMarco Bauzá ThorbrüggeAnton KováčikMarco BiagiAnton TerasmaaMarco BisagliaAnton V. NaumovMarco BortolusAntonella ArestaMarco CordaniAntonella CamaioniMarco Dalla RosaAntonella Caterina BocciaMarco FioreAntonella D’AgostinoMarco G. SchiavoneAntonella D’AnneoMarco NisiAntonella Di SottoMarco PiccoliAntonella OliveroMarco PoianaAntonella PantaleoMarco RoversoAntonella RosiMarco SegattoAntonella ScorzielloMarco VincetiAntonella SgarbossaMarcos Soto-HernándezAntonella SmeriglioMarcus GrimmAntonello SantiniMarek MuriasAntoni SuredaMarek PieszkaAntonia Concetta EliaMarek SkrzypskiAntonia M. Jiménez-MonrealMarek WesołowskiAntonietta GattiMarek WiergowskiAntonietta LiottiMargarida Castro-CaldasAntonín PřidalMargarida DuarteAntonina SidotiMargarita NeganovaAntonino NizzaMargherita MaranesiAntonio BarberisMargreet VissersAntonio BevilacquaMari ChiritoiuAntonio Carrillo-VicoMaria A. AnderssonAntonio CicchellaMaria A. Del Pozo BayónAntonio CillaMaria AlbertiniAntonio Di BartolomeoMaría Ángeles Ferrer AyalaAntonio Díaz-QuintanaMaría Ángeles PeinadoAntonio Doménech-CarbóMaria Angeles RojoAntonio Eduardo Miller CrottiMaria Antonietta DettoriAntonio G. SolimandoMaria Antonietta PanaroAntonio GarciaMaria AtanassovaAntonio GloriaMaría Ávila-GálvezAntonio Gonzalez-CasadoMaria BabakAntónio M. PeresMaria BarretoAntonio MinniMaria Beatriz Durán-AlonsoAntonio Morales De La NuezMaria Carla CraveroAntónio NogueiraMaria Carla MarcotullioAntonio Palumbo PiccionelloMaria Carmela CerraAntonio PortugalMaria Carmo BarretoAntonio Rosales MartínezMaria Chiara MontiAntonio SerratoMaria Cristina CaroleoAntonio Simone LaganàMaria Da Graça Costa MiguelAntonio TirelliMaria Da Graça P. Morgado S. NevesAntonios MakridisMaría De La Luz Cádiz-GurreaAntony C. CalokerinosMaria De Lurdes DapkeviciusAnurag TripathiMaría Delgado EstebanAnwen ShaoMaria Dolores MauricioArabela Elena UnteaMaría Dolores Ortiz-MasiáArafat Abdel Hamed Abdel LatefMaria Dulce EstêvãoArantzazu Santamaria-EchartMaria E. M. AraujoArasu GanesanMaria Eduardo-FigueiraArcady MarkelMaria Eugénia PinaAria BaniahmadMaría F. MontenegroAriana HuditaMaría Figueiredo-GonzálezAriane RozzaMaría GiménezArianna Carolina RosaMaria Giovanna ScioliArianna ScuteriMaria Grazia CerritoArianna VigniniMaria Grazia Di CertoArif KhanMaria GreabuArijit NathMaria Helena FernandesAristidis S. VeskoukisMaria HernandezAristidis TsatsakisMaria IrakliArkadiusz DziedzicMaría Isabel Torres LópezArkadiusz KosmalaMaria J. AlcaldeArmando CardosoMaria J. CrespoArmando Varela-RamirezMaría J. PeralArmando ZarrelliMaría Jesús OrtegaArmandodoriano BiancoMaría Jesús Rodríguez-YoldiArmida Sánchez-EscalanteMaria João BarrocaArmindo Armindo MeloMaria João MartinsArmindo SalvadorMaria João RamalhosaArmond DaciMaria João SousaArno E. LindnerMaría José AlcarazArokia Mariyadoss VijayaanandMaria Josè SisalliArpita ChatterjeeMaria LasalviaArrigo CiceroMaria Lisa GaravagliaArti V. ShindeMaría Luisa Fernández-De CórdovaArtur BeberokMaria Luisa Louro MartinsArtur KhannanovMaría Luisa Ojeda MurilloArtur NosalewiczMaria Luísa SerralheiroArtur SzwengielMaría Luisa Soto MontenegroArturo BevilacquaMaría Luz CouceArturo OrtegaMaría M. Morales Suárez-VarelaAsad JanMaria MaistoAshfaq AhmadMaria Manuela GasparAshutosh BahugunaMaria MartinsAshutosh KumarMaria MycielskaAshutosh TripathiMaria NiarchouAsiya MustafinaMaria P. TsantarliotouAşkım Hediye SekmenMaria Paola NittiAsmaa KhafagaMaría Pérez SerratosaAssunta PandolfiMaria Pia FerrazAstrid HammerMaria Pia RigobelloAstrid KostersMaría Pilar Fernández-MateosAstrid WeißMaria Pina MollicaAsunción MoránMaria Pina SerraAsuncion RocherMaría Rocío Rodríguez ArcosAthanasia VarvaresouMaría Ruiz LinaAthanasios KimbarisMaria RussoAthanasios KoukounarasMaria S. MuntyanAthanasios SalifoglouMaría SerranoAthinoula L. PetrouMaría Shantal Rodríguez-FloresAtish MukherjiMaría Soledad PratsAtsushi IwaiMaria Teresa PretelAtsushi KoikeMaria TikhonovaAtsushi TanakaMaría U. MorenoAttila L. ÁdámMaria V. TurovskayaAuriel A. WilletteMaría Val Toledo LoboAurora M. NedelcuMaria Valeria CataniAvinash P. ManianMaria WallertAvisek MajumderMaria ZychAxel G. GriesbeckMariacristina SiottoAxel H. SchönthalMaria-Eduardo FigueiraAya Naiki-ItoMaría-José ArgenteAzzurra StefanucciMariam GaidBaiba VilneMariana Atena PoianaBalik DzhambazovMariana CastroBaojin DingMarianna A. PatrauchanBaolei JiaMarianna Flora TomaselloBarbara BelleiMarianna MazzaBarbara De FilippisMarianna RaczykBarbara GierlikowskaMarianna SantonastasoBarbara GowerMariano Martínez VázquezBarbara LipińskaMariano OstuniBarbara MerloMariano Walter PertinoBarbara MiroslawMariantonietta Di StefanoBarbara Szeiffova BacovaMariarosaria BucciBarbara TavazziMaricica PacurariBarbara TomaselloMarie Christine CadierguesBarna BalazsMarie Christinne ScherrmannBarry ParsonsMarie Hélène David CordonnierBart De GeestMarie MigaudBartolome SabaterMarie Thérèse CharlesBartolomé VilanovaMarie-Aleth Lacaille-DuboisBaruch SnehMarie-Paule GonthierBasem S. ZakariaMarie-Pierre GolinelliBasheer MannasahebMarija KovačBasini GiuseppinaMarijana Zovko KončićBeata Borowiak-SobkowiakMariko MiyazakiBeata DrużyńskaMarilia Oliveria Fonseca GoulartBeata JasiewiczMarilisa LeoneBeata Kalska-SzostkoMarilyn J. DuncanBeata Kasztelan-SzczerbinskaMarin RojeBeata KuczyńskaMarin ŞenilăBeata NowakMarina BacciBeata OlejnickaMarina CaldaraBeata PajakMarina Garcia-MaciaBeata TarnackaMarina I. GarinBeatrice HeimMarina MariniBeatrice ScazzocchioMarina PaolucciBeatrice SeverinoMarina PiscopoBeatrix AlegreMarina QuartuBeatriz Caballero-GarciaMarina RoginskayaBeatriz I. Vázquez BeldaMarina SantaellaBeatriz PortoMarine CouéBeda HartmannMarinka Mravak StipetićBegoña CerdáMarino PrearoBegoña Ruiz-LarreaMario AbateBe-Jen WangMario DiazBéla JuhászMario FotiBelém Sampaio-MarquesMario G. BianchettiBelén Gómez-GonzálezMario RangoBenedetta MatteiMario Vincenzo RussoBenito A. YardMariola KozłowskaBenjamin CullMarion CurtisBenjamin DelpratMarion EhrichBenjamín FloránMarios G. KrokidisBenjamin J. BurwitzMarios SpanakisBenjamin MaskreyMarisa FreitasBenjamín PinedaMarisa PalazzoBenno WeigmannMarisa VarrentiBernard RoelenMarisol VillalvaBernardo BaldisserottoMaristella GussoniBernardo StutzMarius Emil RusuBernd MoosmannMarius StefanBernhard HuchzermeyerMariusz Aleksander BromkeBernhard RyffelMariusz SikoraBerthold HocherMariusz TrytekBertrand LiagreMark B. HamptonBeth M. ClevelandMark ClemensBhagavathi SundaramMark D. TricklebankBhumsoo KimMark RinnerthalerBianca Elena MasertiMark S. BorjaBianca VezzaniMark S. TaylorBijayalaxmi MohantyMark TarnopolskyBijorn Omar BalzaminoMarkku KeinänenBiju ThomasMarko GerićBilal Ahamad ParayMarko KassBiljana BujanovicMarko SamardžijaBimal GhimireMarkus MandlBin WuMarkus VeltenBing-Lan LiuMarlena SzalataBingwen XiMarouane BaslamBin-Huei ChenMars G. SharapovBinxing LiMarta Alegret JordaBirandra K. SinhaMarta Anna KowalikBirgit Lichtenberg-KraagMarta GaburjákováBiserka RelicMarta LadányiBlagovesta PopovaMarta Liszka-SkoczylasBlanca Hernández-LedesmaMarta LlansolaBlanca MolinsMarta MarmiroliBo ÅkerströmMarta MartínezBo HuMarta MenegazziBo WangMárta M-HamvasBoel De PaepeMarta PerezBogdan KontekMarta Prieto VicenteBogdan SevastreMarta PutrinšBogdan SoceaMarta RachelBogumil BryckiMarta SarkozyBogumiła PilarczykMarta SauraBojan ButinarMarta Seco-CerveraBoleslaw KarwowskiMarta SochockaBonaccorsi Di Patti Maria CarmelaMarta Tsirigotis-ManieckaBonetti AlessandraMartha Gabriela Gaxiola-CortesBoon-Seng WongMartin ČernýBor Luen TangMartin F. LavinBoris ErshovMartin HimlyBorislav AngelovMartin JaburekBoros MihályMartin Johannes PotgieterBor-Ran LiMartin KuentzBorut PoljšakMartin MetzgerBouzas CristinaMartin PecBo-Young HongMartin RasporBozena MuszynskaMartin SchepelmannBrandon Lucke-WoldMartin SvobodaBrandon Mark GassawayMartin WeissBranislav KuraMartina BortolamiBreda Jakovac-StrajnMartina CebovaBrenda A. Silva-EspinozaMartina HöcknerBrendan M. DugganMartina MeinkeBrent A. BakerMartina Paumann-PageBrent SieskyMartina SmolićBrian J. DayMartina ValachovičováBrian Jay KerrMary Beth MadonnaBrian JonesMary Cano-SarabiaBrian McdonaghMary TaubBrian OliverMaryam N. Al-NasserBrigitta TóthMaryline Magnin-RobertBrittney HarringtonMarzena MackowiakBruna LarattaMarzena Mogielnicka-BrzozowskaBruno BottaMarzena WójcikBruno CerraMaša KandušerBruno ChrcanovicMaša Knez MarevciBruno FigadereMasafumi FunamotoBruno GlaserMasahiko TsuchiyaBruno MeloniMasahiko YamaguchiBruno MezzettiMasakazu SugishimaBruno SousaMasaki OkamotoBruno StefanonMasami NozakiBruno TrimarcoMasamitsu KanadaBryan Andrew KillingerMasanori HonshoBryan MathisMasanori KatakuraBulent MutusMasanori Masanori HijiokaBurkhard KleuserMasashi KawamiByron AsimakopoulosMasayuki FujitaByung Eui KimMassimiliano RuscicaByungsuk KwonMassimiliano TognoliniC. Michael GreenliefMassimo ColettaCaitlyn BowmanMassimo CorsaliniCamelia Sorina StancuMassimo D’ArchivioCamelia UngureanuMassimo GrilliCamillo La MesaMassimo IorizzoCamilo Dias Seabra PereiraMassimo NabissiCampbell H. ThompsonMatea Nikolac PerkovicCarl Friedrich ClassenMateja GermCarla CaddeoMateja J. PrimožičCarla CeoloniMathews VargheseCarla FerreriMathieu F. CellierCarla GentileMathilde Body-MalapelCarla SilvaMats ErikssonCarla VillaMatteo GoldoniCarlo CorinoMatteo PaciniCarlo CosentinoMatthaios P. KavvalakisCarlo GenoveseMatthew D. StachlerCarlo LajoloMatthew DodsonCarloalberto PettiMatthias ElsnerCarlos Alfonso Álvarez-GonzálezMatthias Hardtke-WolenskiCarlos Alfredo Sandoval CastroMatthias NéesCarlos Andres Galan-VidalMatthieu MillionCarlos GarcíaMatthieu Pierre PlatreCarlos M. CorreiaMattia Di NunzioCarlos Pinto GomesMattu ClaraCarlos R. FigueroaMaura LodoviciCarlos R. TirapelliMauricio Alcolea PalafoxCarlos RosadoMaurício Temotheo Temotheo TavaresCarlos Sandoval-CastroMaurizio BadianiCarlota Gómez-RincónMaurizio CammalleriCarlotta GirominiMaurizio ForteCarme GüellMaurizio MandalàCarmela Maria MontoneMaurizio MargaglioneCarmela SantangeloMauro AngelettiCarmelina SpanòMauro ColucciaCarmelita FrondozaMauro IulianoCarmelo Maria MusarellaMauro LombardoCarmen AvaglianoMaxime RobinCarmen C. LlenaMaya GunchevaCarmen CuadradoMayada Ragab FaragCarmen Del Toro SánchezMayumi KomineCarmen FestaMayya PetrovaCarmen Garcia-JaresMayya RazgonovaCarmen Lăcrămioara ZamfirMd AdnanCarmen López SánchezMd Badrul AlamCarmen M. VázquezMd. Mezanur RahmanCarmen Martin CorderoMedha PriyadarshiniCarmen RamírezMehmet M. AltintasCarmen Regla VargasMehtap SahinerCarmen SaezMei-Chin LuCarmen SotoMelanie Ricke-HochCarmine RoccaMelinda FogarasiCarmine SummoMelinda K. DuncanCarola CavalloMelissa FathCarolina Alquezar-BurilloMelissa PageCarolina Sánchez-RodríguezMelissa PuppaCarolina TamargoMeng-Tsan ChiangCaroline Cieniewski-BernardMengxing LiCaroline MarconMercè LluganyCarsten CarlbergMercè Pallàs LliberiaCassie S. MitchellMercedes GarayoaCatarina Maria QuinziiMercedes Garcia-GilCatello Di MartinoMercedes Vázquez-EspinosaCaterina LiciniMeriel G. JonesCaterina PeggionMi Kyeong LeeCaterina PipinoMichael AlexisCaterina SquillaciotiMichael AzainCatharina Sagita MoniagaMichael BlankeCatherine ChanMichael Brad StraderCatherine CookMichael Bruce JarstferCatherine JusteMichael C. SeedsCatherine MounierMichael CalcuttCatherine YzydorczykMichael DaviesCatia ScassellatiMichael DeschenesCavar ZeljkovicMichael E. BoultonCecilia BrunettiMichael EskinCecilia FaraloniMichael HipplerCecilia NunesMichael KundiCédric DelporteMichael LindingerCeferino Carrera FernándezMichael MorganCéline SterMichael MurkovicCelio Geraldo Freire-De-LimaMichael PoteserCerasela Elena GîrdMichael R. KnittlerCesar BorlonganMichael RothCésar C. Leyva-PorrasMichael SticherlingCésar Fernández-SánchezMichael WempeCezar ComanescuMichaela ŠiklováCezary A. KwiatkowskiMichał MączewskiCezary WatałaMichał OtrębaChabaco ArmijosMichal Shoshkes-CarmelChad H. StahlMichał Stefan LachCháfer-Pericás ConsueloMichał ŚwiecaChahrazade El-AmriMichal ZemanChandrakala Aluganti NarasimhuluMichel HavauxChang ChenMichela AspertiChang Won ChoiMichela FerrucciChang Yeon YuMichela GiulianoChang-Gue SonMichela VerniChangqi LiuMichele CiullaChangquan LingMichele MaffiaChangwei HsiehMichele NavarraChang-Wei HsiehMichele PritchardChangwon YangMichèle SalmainChangyu ZhouMichele SamajaChantal HoueeMichelle Ji Yeon YooChao HeMichelle YooChaofan LiMichiaki YamashitaChao-Hui FengMichio IwaokaChaokai ChangMiguel AlcarazCharalampos KarantonisMiguel Angel Garcia-AlvaradoCharalampos ProestosMiguel Ángel PrietoCharalampos SiristatidisMiguel LaderoCharles D. RiceMiguel Navarro-AlarconCharles NortonMiguel Rebollo-HernanzCharlotte LawsonMiguel RomeroCharlotte Von GallMiguel TeixeiraCharng-Cherng ChyauMihaela HostiucChavis KetkaewMihaela Radu (Balas)Chen ChuMihaela SkrtChen Huei LeoMihaela TurtoiChenchen ZhaoMihai CenariuCheng-Tien WuMihai RaduCheng-Yang HuangMihail Lucian BirsaCheng-Yoong PangMihail MitovChenxu YuMihi YangCheol-Su KimMikael BjornstedtChetan KeswaniMike WilliamsonChhanda BoseMikel San-JuliánChi Keung LamMikhail GolovkoChi LiMikhail M. Vorob’EvChia-Hsien FengMikhail V. DubininChia-Hung KuoMikko NikinmaaChia-Hung LeeMikko O. LaukkanenChia-Hung YenMiklós GeisztChiaki YoshikawaMiklós MézesChia-Min LinMiklós NagyChianju JongMiklos PeterfyChiara AlisiMilad HadidiChiara B. M. PlataniaMilagros BuenoChiara BernardiniMilagros MedinaChiara FoglieniMilan ČížChiara Maria MottaMilan SkalickýChiara NovielliMilan UrbanChiara PanicucciMilica AćimovićChiara RossoMilica MarkovicChiara ValoriMilica PešićChiara VillaMilos R. FilipovicChi-Chen LinMilva PepiChi-Chiu WangMin JinChien-Chung HuangMin XieChien-Chung YangMina LeeChien-Teh ChenMing ChenChiharu OtaMing ZhangChih-Chuang LiawMing-Che LiuChih-Hao WangMingfu ChenChih-Hsin TangMing-Kuei ShihChih-Li LinMinglei GuoChih-Wen ShuMingquan GuoChih-Yao HouMingxing LeiChi-Ming WongMing-Yi ShenChing-Feng WengMingyi WuChing-Hui YehMing-Yii HuangChing-Jin ChangMing-Yu LungChing-Yi ChenMin-Ho OakChin-Kun WangMin-Sik KimChi-Rei WuMinyoung KimChitra VenugopalMioara BercuChiu-Li YehMiodrag JanicChris YunMircea Adrian OroianChrishan RamachandraMircea TampaChristelle GuibertMirela KopjarChristian BarbatoMirela PlaninićChristian BergaminiMiriam GaggianesiChristian BleilevensMirian PateiroChristian Cortés-RojoMiriana DuranteChristian JungnickelMirjana JerkicChristian L. LaboisseMirko ManchiaChristine BoschMirko ManettiChristine RondaninoMiroslav BarančíkChristine WinterbournMiroslav ChovanecChristoph FaschingerMiroslava PožgajováChristoph OttoMiroslaw JablonskiChristoph ReinhardtMiroslaw JanowskiChristophe GaillochetMirosław KrośniakChristopher P. MattisonMirosław WyszkowskiChristopher R. M. AsquithMirosława ChwilChristos KontogiorgisMiryam Amigo-BenaventChristos PapaneophytouMitchell C. ColemanChristos PappasMitsuru HarukiChrysavgi GardeliMitsuru NakazawaChuang-Rung ChangMizue TeraiChuangyu WenModestas ŽiliusChuen-Mao YangMohamed AbdelwahabChuleui JungMohamed Ali IbrahimChun GuoMohamed SolimanChun-An ChengMohammad Azam AnsariChunbin ZouMohammad HossainChundong YuMohammad NajlahChung Y. HsuMohammad Rehan AjmalChung-Hsin WuMohammad SadraeianChung-Hwan ChenMohammed AkbarChung-Ming ChenMohammed EmranChun-Lin LeeMohammed S. RazzaqueChunling HuangMohd AdnanChun-Ping LinMohd RaufChunqi GaoMohsin JafriChunwoo YangMoisés Martínez VelázquezChunying LiMolly YaoChunzhang YangMona BoazCicero ArrigoMong-Heng WangCinzia FabriziMonica CurròCinzia ForniMonica De CaroliCinzia FranchinMonica FlorescuCinzia MontemurroMonica GalloCinzia RapinoMonica GelzoCiro IaccarinoMonica GomaraschiCiro ImbimboMonica NardiClaire M. KellyMonica R. LoizzoClara GrossoMónica Rodríguez García-RiscoClaudia AndlMonica Rosa LoizzoClaudia Di GiacomoMonica ValentovicClaudia FotiMonika CzerwińskaCláudia M. BotelhoMonika KassayováClaudia MartiniMonika KijewskaClaudia MatteucciMonika KosmalaClaudia PereiraMonika Les̈KiewiczClaudia SayerMonika M. GladkaClaudio BucoloMonika SommerhalterClaudio CantiniMonika WasilewskaClaudio De SimoneMonokesh Kumer SenClaudio FerranteMontecucco FabrizioClaudio HumeresMontserrat Esteve RàfolsClaudio LombardelliMontserrat MaríClaudio LuchiniMontserrat Mitjans ArnalCláudio MaiaMontserrat SoléClaudio SantiMoon Nyeo ParkClaus P. W. ZebitzMorena GabrieleClinton WebbMoreno BurgosColin J. SucklingMorris ManolsonColin W. MacdiarmidMosiewicz JerzyCollynn WoellerMotoi KikusatoConcepció AmatMotonari TsubakiConcepción GarcíaMoushumi GhoshCong PengMudit TyagiConor MccleanMugurel Constantin RusuConsuelo Cháfer-PericásMuhammad Ahsan AsgharCoral SanfeliuMuhammad Iqbal R. KhanCorina CarrancaMuhammad Jawad NasimCornelia BalaMuhammad JunaidCornelia MirceaMuhammad Shahid Riaz RajokaCorrado RizziMuhammad SyafrudinCorrea-De-Araujo RosalyMuhammad Wahab AmjadCortés-Castell ErnestoMumtaz AnwarCosima D. CalvanoMunekazu YamakuchiCosmas NathanailidesMurali DamaCostantino BalestraMustafa ShukryCostantino MancusiMustapha Cherkaoui MalkiCostantino PaciollaMuthaiah ShellaiahCostas E. StathopoulosMyeong-Cheoul ChoCourtney HouchenMyoung-Hwan ParkCrisalejandra Rivera-PérezMyron ChristodoulidesCriscuolo ElenaMyung-Bok WieCristian BaicusMyungsoo JooCristian BonviciniNabil ElsheeryCristian Del Bo’Nada BožinaCristian O’FlahertyNadejda I. RechkunovaCristian PîrvuNadezda ApostolovaCristián Sandoval-AcuñaNadezhda GolubkinaCristian StătescuNadia BoussettaaCristina CalejaNadia CattaneCristina CarresiNadia RegaCristina ComanNafees A. KhanCristina GluhovschiNagavendra KommineniCristina Gutierrez-SanchezNagendra Kumar KaushikCristina LlorenteNahla S. El-ShenawyCristina M. L. CarvalhoNakao KuboCristina MaccalliniNałȩcz-Jawecki GrzegorzCristina Manuela DrăgoiNalladurai KaliyanCristina Mihaela NicolescuNam Deuk KimCristina NavarroNanjangud V. Anil KumarCristina SatrianoNaoki TakahashiCristina SgherriNaoki TanakaCristina SoaresNaoya KishikawaCristina SobacchiNaoyuki TaniguchiCristina SuñolNaphtali SavionCristina TravelliNarasimham ParinandiCristina VinciNarasimman GurusamyCristobal AguilarNarayanankutty ArunaksharanCristobal De Los RiosNarcis TribulovaCristobal EspinosaNaroa KajarabilleCristobal MirandaNarsa M. ReddyCsaba HanczNassim MoulaCsaba SzaboNatalia A. MuralevaCsandra SobocanecNatalia ArendarczykCyrielle BillonNatalia BaranCyril NicolasNatalia BelosludtsevaCyrille RichardNatalia IssaevaCyrus MotamedNatalia Ivanovna AgalakovaDae Youn HwangNatalia JatkowskaDaeui ParkNatalia Martínez-GilDagmara BaczyńskaNatalia V. GulyaevaDagmara Wróbel-BiedrawaNatalie LenardDahn ClemensNatalija FilipovicDaiji EndohNataliya KolosovaDaisuke SeoNatasa MilicDaisuke TodakaNatassa PippaDalia El KhouryNatércia TeixeiraDalia M. KopustinskieneNathan M. D’CunhaDalibor KodrikNathaniel SzewczykDâmaris SilveiraNavneet DograDamian SkrypnikNayden G. NaydenovDan ChenNazareno PaolocciDana CairnsNeena PhilipsDana CucuNeha GargDana Elena PopaNela MalatestiDanica JovićNeli Kinga OlahDaniel ArcanjoNemat AliDaniel B. Kim-ShapiroNeven ZarkovicDaniel C. MorrisNguyet-Thanh Ha DuongDaniel ElbirtNgyen QuangDaniel HeruthNicholas G. JamesDaniel Jacobo-VelazquezNicholas HengDaniel KamińskiNicholas T. BelloDaniel KönigNicholas T. BroskeyDaniel LinsemanNicholas V. C. RalstonDaniel M. KaminskiNicola ArmstrongDaniel MlocickiNicola CirilloDaniel P. PotaczekNicola IngramDaniel SmržNicola MicaleDaniel VaimanNicolae GicaDaniela BeghelliNicolas DiotelDaniela CaccamoNicole BarraDaniela CarlisiNicole SchuppDaniela DamianiNicoleta BadeaDaniela GabbiaNicoletta ZoppiDaniela GiacomazzaNidhi Jalan-SakrikarDaniela ImpellizzeriNiels Henrik BuusDaniela MarascoNikhlesh SinghDaniela MokraNiki L. ReynaertDaniela QuaglinoNikita TsvetovDaniela StrenkertNikol SulloDaniela Valeria MinieroNikolaos G. KostomitsopoulosDaniele Bobrowski RodriguesNikolaos GeorgopoulosDaniele Filippo CondorelliNikolaos LabrouDaniele MasaroneNikolaos SiafakasDaniele SolaNikolas J. HodgesDaniele TibulloNikolay Ul’YanovskiyDaniele TomassoniNikole ByrneDanielle GreenbergNikos MargaritelisDaniil OlennikovNils BömerDanila CianciosiNilva Kazue SakomuraDanila LimauroNina DickerhofDanina MunteanNina TumosaDanuta Januszkiewicz-LewandowskaNingning WangDanuta JaworskaNino RussoDany Munoz-PintoNirakar SahooDaocheng WuNitikaDapeng WangNoah MoruzziDaret St. ClairNobuaki OkumuraDaria MelnikovaNobuhiro SuzukiDarío Acuña-CastroviejoNobuyuki KimuraDario BrunettiNoelia DuarteDario Di SilvestreNoélia DuarteDario Domenico LofrumentoNoemi Rotllan VilaDario FinazziNoreen F. RossiDario KremerNorihiko OhbayashiDario PaoloNorihisa KatoDario RuscianoNorio SogawaDariusz KucharczykNoriyuki MiyoshiDariusz NowakNorman Scott LitofskyDarya TarbeevaNoura DosokyDasha MihaylovaNozik EvaDasheng LuNunzio CardulloDavid A. GellObie FarobieDavid Allan ButterfieldOctavio Paredes-LópezDavid Arráez RománOfélia AnjosDavid BeversdorfØivind BerghDavid C. IrwinOke GerkeDavid E. StecOk-Nam BaeDavid GreenhalghOksana SherstnevaDavid GrynspanOksana V. SytarDavid HarmonOlaf MerkelDavid HoodOldrich LapcíkDavid HoogewijsOleg MatusovskyDavid J. McgeeOleg N. TimofeevDavid LaightOlga A. RozentsvetDavid LawOlga AfanasievaDavid Lozano-PaniaguaOlga EscuderoDavid M. ConradOlga LuzinaDavid NelsonOlga MartinDavid PereiraOlga ProtchenkoDavid RossOlga RedinaDavid ŠilhaOlga Rojo-PovedaDavid WalkerOlga TsigkouDavid WilliamsOlimpia CarrerasDavide BarrecaOlimpia VincentiniDavide LauroOlivera PoliteoDawei GaoOlivier SorgDawid StefaniukOlivier VallonDebabrata BanerjeeOluyinka A. OlukosiDebasish BandyopadhyayOmar CauliDebora FontaniniOndrej SlanarDebora VillañoOndrej VasicekDebottam SinhaOren PleniceanuDeepak Kumar KhajuriaOriana TrubianiDeepika AwasthiOrietta PansarasaDelia MezzanzanicaOrly Lacham-KaplanDelia MunteanOrsolina PetilloDemetrios ArvanitisOrtwin NaujokDenis BourgeoisOsamu KaminumaDenis N. SilachevÓscar Lorenzo GonzálezDenise G. HemmingsOscar NunezDennis CladisOtilia BobisDerek ParsonageOyuna KozhevnikovaDerval Dos Santos RosaOzgul GokDesiree WandersP. Hemachandra ReddyDevy DeliyantiPablo Fernández-TussyDhananjayan VasuPablo Hernansanz-AgustínDhimiter BelloPablo MierDhiraj P. MuralePablo MurielDi ShiPaloma Fernández VarelaDiaa YoussefPamela MaherDiana AmanteaPamela SenesiDiana Andreia Tavares PintoPamela VignoliniDiana AntalPanagiotis D. KatsoulosDiana Cláudia PintoPanagiotis EfentakisDiana CostaPanagiotis J. SkourasDiana Crus-TopetePanagiotis KandylisDiana De SantisPanagiotis KoulouvarisDiana Hernandez-RomeroPanagiotis SimitzisDidac SantesmassesPanagiotis TsiakanikasDidier F. PisaniPankaj AttriDiego A. MorenoPaola BagnoliDiego ColomboPaola CortiDiego CotellaPaola CostanzoDiego Luís CostaPaola Di PietroDietmar EnkoPaola FiniDietmar FuchsPaola LenziDimitra J. DafereraPaola ManiniDimitrina Zheleva-DimitrovaPaola MarcolongoDimitrios BikiarisPaola MontoroDimitrios GalarisPaola NavarreteDimitrios KanelisPaola PalestiniDimitrios KouretasPaola PiomboniDimitrios StagosPaola TedeschiDimitris A. GougoulisPaola TuranoDimitris EmfietzoglouPaola VendittiDimitris K. KantasPaolo ArosioDimitris KletsasPaolo BergamoDimitris P. MakrisPaolo BonilauriDimitris TsoukalasPaolo ContiDimosthenis ChochlakisPaolo IovienoDini IrenePaolo MondolaDinora F. González EsquivelPaolo PastorinoDionisios GasparatosPaolo ScudieriDipankar RoyPaolo SilacciDirk MetzlerPaolo TotiDirk W. LachenmeierParameswaran G. SreekumarDjanaguiraman MaduraimuthuParamjit TappiaDmitriy AtochinParaskevi KritsiligkouDmitry B. ZorovParaskevi MitliagkaDmitry KarpovParmjit S. JatDo-Hee KimPartha MukhopadhyayDohoon KimParthasarathi RamakrishnanDolores González De LlanoPascal ReyDomenica MangieriPaschalis-Thomas DouliasDomenico De RasmoPasquale AvinoDomenico LioPasquale CrupiDomenico MontesanoPasquale EspositoDomenico TafuriPasquale PignatelliDomenico TricaricoPasqualina BuonoDominik KmiecikPatrice X. PetitDominik R. HaudenschildPatricia BoyaDominika GłąbskaPatricia CuervoDominika ZającPatricia Seoane-CollazoDonald MessnerPatrícia SeverinoDonatella Degl’InnocentiPatricio Fernández-SilvaDonatella DuraccioPatrick BradshawDonatella VerbanacPatrick GeraghtyDong MengPatrick HornDongbao ChenPatrick SipsDonggwan KimPatrick VideauDong-Joo CheonPatrick WeydtDongmin LiuPatrizia GuidiDorit AvniPatrizia LeoneDoron ShkolnikPatrizia PicernoDorota DymkowskaPatrizia Polverino De LauretoDorota Gołąbek-SulejczakPatrizia RomualdiDorota LechniakPatrycja KleczkowskaDorota NowakPatrycja MichalskaDorota Rutkowska-ZbikPaul CopelandDorota SulejczakPaul G. FurtmüllerDorota Tichaczek-GoskaPaul GardDorota Tomaszewska-ZarembaPaul J. ThornalleyDorota WojtysiakPaul JoyceDorota WrześniokPaul MurrayDorota ŻyżelewiczPaul PrenzlerDorothee WeihrauchPaul RöschDouglas SpitzPaula C. CastilhoDouny CarolinePaula SilvaDragana Mitić-ĆulafićPaula VerissimoDragomir YankovPaulina DonosoDragos SerbanPaulina DumnickaDrew NassalPaulina KoczurkiewiczDubravko HabekPaulo Eduardo MunekataDulcenombre Gómez-GarrePaulo J. PalmaDunja ŠamecPaulo José Gomes CoutinhoDurai SellegounderPaulo PiresDušan PalićPaulo SantosDušan ŠuputPavana RottiEberhard SchlickerPavel FeduraevEdivaldo Herculano Correa De OliveiraPavel KosinaEdmundo Rosales-MayorPavel MakarevichEdoardo Marco NapoliPavel V. AvdoninEdouard GerbaudPavlina DolashkaEduardo Domínguez MedinaPawel Antoni KolodziejskiEduardo Fernández-MartínezPaweł GaćEduardo Fuentes QuinterosPaweł GlibowskiEduardo GuzmánPawel KafarskiEduardo Molina-JijonPaweł KonieczkaEduardo OsunaPaweł PaśkoEduardo RialPawel PohlEduardo Valencia-CanteroPayaningal R. SomanathEdvardas DanilaPedro AraujoEdward BahnsonPedro BullonEdward H. SharmanPedro D. VazEdward N. HarrisPedro Diaz-VivancosEdward RojPedro FernandesEdwin D. LephartPedro Ferreira-SantosEdyta MakuchPedro LaxEdyta MolikPedro Martínez-GómezEdyta SierkaPedro Nunez-AbadesEdyta SymoniukPedro RomãoEfigenia Montalvo GonzálezPedro TalhinhasEfthimia AntonopoulouPeggy MarconiEgeria ScodittiPeichao ChenEgor Y. PlotnikovPenelope AnguileraEhab AbourashedPenelope Perkins-VeazieEhab H. SarsourPeng LiEichenlaub-Ritter UrsulaPeng ZouEiichi KumamotoPengfei ZhangEiichi SatoPengzhan LiuEiji GotoPer Mårten HägglundEiji YoshiharaPernille Tveden-NyborgEikan MishimaPetar OzretićEirini GrapsaPéter Ábrányi-BaloghEleftheria GalatouPeter AbujaEleftherios M. BonosPeter BališElena BerezhnayaPeter C. GainesElena BoggioPeter CelecElena Del FaveroPeter Christopher KonturekElena DeligianniPeter H. J. Van Der VoortElena EfremenkoPeter John JervisElena Falqué LópezPeter KubatkaElena GiordanoPeter KubiszElena KaschinaPeter LorenzElena KosenkoPeter MassányiElena LevantiniPeter MccourtElena Peñas PozoPeter ParsonsElena PohlPeter PolčicElena TomsikPeter RoseElena TorrieriPéter SzentesiElena V. SvirshchevskayaPeter TomčíkElena VismaraPeter VeranicEleni AbrahamPeter F. SuraiEleni MavrogonatouPetko DenevEleni TsantiliPetr HenebergEleonora MarianPetr MlejnekEleonora NapoliPetra Peharec ŠtefanićEleonora PagnottaPetras Rimantas VenskutonisEleonora PorcuPetros P. PetrouEleonora ScarlataPeyman RezaieElia RanzatoPhil AyersElie H. MotulskyPhilip I. AaronsonElina PeltomaaPhilipp S. LangeElio PizzoPhilippe De DeurwaerdereElisa CairrãoPhiwayinkosi V. DludlaElisa NutiPhoebe X. QiElisa PassagliaPhotini MylonaElisa ZavattaroPia Lopez-JornetElisabeta BădilăPier Nicola SergiElisabeth PinartPierangelo FreschiÉlisabeth TraiffortPierangelo LuporiniElisabetta CasalinoPierfrancesco CerrutiElisabetta CatalaniPierluigi StipaElisabetta GiudicePiero SestiliElisabetta MariniPierre TennstedtElisabetta ProfumoPierre VacherElisabetta TostiPierre-Yves Collart-DutilleulElite PossikPieter De LangeElizabeth Bartolak-SukiPietro GareriElizabeth C. CottrellPiia KarisolaElizabeth C. LedgerwoodPilar ArrueboElizabeth Carvajal-MillanPilar D’OconElizabeth FernandesPilar Fernández MateosElizabeth G. IngulliPing YaoElizabeth J. JohnsonPing ZhouElizabeth L. RyanPing-Chung KuoElizabeth Podlaha-MurphyPing-Hsun WuElizabeth S. FernandesPing-Hui TsengEllen HertzmarkPing-Shan LaiEllen NiederbergerPiotr BrągoszewskiElrashdy M. RedwanPiotr DobrowolskiElsa C. ChanPiotr KusElsa M. GonçalvesPiotr KuśElsa UribePiotr LatochaElvira CrescenziPiotr MichelElwira ChrobakPiotr OstaszewskiElżbieta BudziszPiotr P. WieczorekElzbieta JandaPiotr StefanowiczElżbieta Łodyga-ChruścińskaPiotr SzwedaElżbieta Lorenc-KociPiotr WlaźElżbieta MillerPo-Hsien LiElzbieta SalinskaPorfirio NavaElżbieta Studzińska-SrokaPouwedeou Mouloumdema PotchoElżbieta Wałajtys-RodePo-Yuan KeEmad Al-DujailiPrabhat C. GoswamiEmanuela BerrinoPrachi UmbarkarEmanuela EspositoPramit ChowdhuryEmanuela MazzonPramod GopalEmanuela ZanardiPrasanna NeelakantanEmanuele BernardinelliPrasanth PuthanveetilEmanuele D’AmicoPrashant KaushikEmanuele PapiniPraveen KogantiEmiliana GiacomelloPrem KushwahaEmiliano BediniPriit VäljamäeEmiliano LaudadioPriyanka RayEmilie WidemannProsper N’GouemoEmilio Álvarez-ParrillaPrzemysław KosobuckiEmilio CervantesPrzemysław Ł. ZalewskiEmilio MartinesPrzemysław PłonkaEmilio SabiaPuran S. BoraEmma BorrelliQian XuEmmanouil N. KokkinakisQiang FuEmmanouil PapaioannouQi-En WangEmmanuel BourdonQifei LiEmmanuel BrouilletQin FuEmmanuel Obeng-GyasiQingyi TongEmmanuel PanterisQinqin PuEmmanuel RichaudQiong GuEmmanuele CrespanQiwei QinEmoke PallQixiang ShaoEnbo MaQuan ZhongEncarnacion Medina-CarmonaRadmila PavlovicEndre SulyokRadoslaw DrozdEng Shi OngRadosław KowalskiEnrico BraccoRadosław KujawskiEnrico FinottiRadu C. RacovitaEnrico NovelliRadu Claudiu FierascuEnrico PierantozziRaelene PickeringEnrico RizzarelliRafael BargielaEnrico Vito PerrinoRafael Guillén-BejaranoEnrique Domínguez-ÁlvarezRafael LeonEnrique GomezRafael Maria PrietoEnrique J. BlancoRafael Prado-GotorEnrique RocheRafael Salto-GonzalezEnzo Maria VingoloRafał FrańskiErcan BursalRafal KretowskiEric A. DeckerRafał StarzyńskiEric A. EppingRaffaele BoniEric BastosRaffaele FrazziEric BattagliaRaffaella BoggiaEric BeyerRaffaella BranciariEric BoncompagniRaffaella ColomboEric D. HamlettRaffaella RebucciEric HallermanRaffaella RossiEric PeeplesRaffaella SorrentinoEric S. PeeplesRaghavendra PalankarErica BazzanRahul SharmaErica CostantiniRaimundo Fernandes De Araújo JúniorErik BeckmannRais AnsariErik BehringerRajeshwary GhoshErik KleinRajinder DawraErika Bevilaqua RangelRajiv DahiyaErika BujnaRalf BraunErika CioneRalf-Erik WellingerErika CottoneRaluca Șuică-BunghezErika PalmieriRam Kumar SelvarajuErin G. Reed-GeaghanRam PrasadErin NorrisRamana VakaErnesto QuesadaRamar ThangamErshuai ZhangRamunas AntanaitisErsilia AlexaRaphael De PaivaErtan YildirimRaphaele De La TorreErwan GalardonRaquel Moreno LoshuertosErxi WuRaquel OlíasEryn L. WerryRaquel P. F. GuinéEsteban C. GabazzaRasa BanieneEster GuausRatha MahendranEswar ShankarRaul Hernandes BortolinEszter DuczaRavi P SahuEthan AndersonRavindran Caspa GokulanEttore VarricchioRayan Khaddaj MallatEugene Haydn WaltersRaymond Scott DuncanEugene P. MazzolaRebeca CruzEugenia BezirtzoglouRebecca L. CharlesEugenio MartelliRebecca OberleyEugenio MiguelReem T. AtawiaEugenio RagazziRegiane Rodrigues Dos SantosEugeniusz Ryszard GrelaRegina Fölster-HolstEun Jeong ParkRegina M. DayEun Joo ChungRégis GuieuEunhee HanReiko AkagiEun-Hye JoeReinhard KalbEunice BacelarRéjean CoutureEun-Kyung KimRéka MizseiEunyoung HaRemigius ChizzolaEva DražanováRemigiusz BąchorEva FalchRena GorovitsEva Fischer-FodorRenata FrancikEva M. Medina-RodríguezRenata Jurišić GrubešićEva Mischak WeissingerRenata KrupaEva SzegöRenata Matraszek-GawronÉva SzökőRenata PerlikowskaEva TvrdáRenata Raina-FultonEvangelia VouvoudiRenata Rucińska-SobkowiakEvangelos DaskalakisRenata Walczak-JędrzejowskaEvgenia MitsouRenato B. PereiraEvgenii PlotnikovRenato PradoEvgeny BlagovechtchenskiRenpeng DuEvgeny LodyginRenuka T. MenonEvguenia BekmanRevathi ShanmugasundaramEwa GlowinskaRex FitzgeraldEwa GondekReza KiaEwa Hanus-FajerskaRiaz UllahEwa Joanna Hanus-FajerskaRicardo D. MorenoEwa MajewskaRicardo De MesquitaEwa Olchowik-GrabarekRicardo N. PereiraEwa Szczepanska-SadowskaRiccardo AmoratiEwa TomaszewskaRiccardo Carlo NatoliEwa Toporowska-KowałskaRiccardo IentileEwelina HallmannRiccardo MavigliaEwoud B. CompeerRiccardo PrimiFabián AcuñaRichard DucatelleFabiana MouraRichard M. NilesFabien P. BlanchetRichard RussellFabio CiccaroneRichard Steven PappasFabio GalvanoRichard W. MankinFábio Rodrigues Ferreira SeivaRichard WagnerFabio SciubbaRicheng ChianFabio VirgiliRita GhoshFabio VivarelliRita Nogueira-FerreiraFabrizio CartaRita Payan-CarreiraFabrizio Dal PiazRita PerriaFabrizio DamianoRita PolitoFabrizio GelminiRita RezzaniFabrizio GentileRitin SharmaFadi KhasawnehRob SiebersFadia YoussefRobert AncuceanuFaiyaz ShakeelRobert BiczakFan KelongRobert ChleboFang WangRobert DaileyFatima CerqueiraRobert F. CasperFatima MartinsRobert J. BrownFátima NogalesRobert J. WalkerFavián TreulénRobert NowakFederica BlandoRobert OstrowskiFederica CioffiRobert PazdroFederica CirilloRobert SochaFederica FogacciRobert T. MeansFederica FregnanRoberta ForlanoFederica IanniRoberta FuscoFederica RivaRoberta GazianoFederico CorlettoRoberta GonnellaFederico GalvagniRoberta ImperatoreFederico HerreraRoberta MasellaFedor SeverinRoberta MoschiniFedora GrandeRoberta PalmourFei ChenRoberta SalaroliFei HeRoberta T. HoskinFelice MastroleoRoberto CampagnaFelipe Martinez-PastorRoberto CarnevaleFelisa Reyes-OrtegaRoberto ChiarelliFelix BeckerRoberto CiarciaFelix ClanchyRoberto CiccorittiFélix Javier Jiménez-JiménezRoberto Di MarcoFelix NitschkeRoberto GramignoliFeng DongRoberto RuiuFeng LiuRobin J. TyackeFengjie SunRobin Lewis CooperFeng-Lin YenRodica BălașaFengqing YangRodica DinicăFengyun MaRodney D. BrittFerdinando FranzoniRodney G. BowdenFerdinando NicolettiRodrigo Herrera-MolinaFerdinando PaternostroRodrigo Moore-CarrascoFerenc DomokiRodrigo TroncosoFerenc GallyasRodrigo ValenzuelaFernanda AmicarelliRoksana MarkiewiczFernanda FidalgoRoland UlberFernando Cordeiro RaposoRolf TeschkeFernando Gandía-HerreroRoman MezencevFernando J. GonçalvesRomana FatoFernando LidonRomeo RomagnoliFernando NovioRon MittlerFernando Peña VegaRonald J. WongFilip SedlicRonan MurphyFilip ŠupljikaRong ZhouFilipa A. VicenteRosa CarotenutoFilipa S. ReisRosa DireitoFilippo BiamonteRosa DivellaFilippo MiglioriniRosa María García-GarcíaFilippo RenòRosa Maria Mateos BernalFiona M. CampbellRosa PalmeriFiore Pasquale NicolettaRosa PerestreloFiorentina RoviezzoRosa RoyFirdos Alam KhanRosa SuadesFlávia Aparecida GraçaRosalba SiracusaFlavia Carla MeottiRosalba Troncoso-RojasFlavia MerigoRosalia CrupiFlora N. BalievaRosália SáFlorent BusiRosalinda Guevara-GuzmánFlorentina Monica RadulyRosângela Da Silva LaurentizFlorentina-Daniela MunteanuRosanna Di PaolaFlorian GembardtRosanna MallamaciFlorian GesslerRosaria IngrassiaFornai MatteoRosaria VarìFotini LamariRosario AmmendolaFrancesca ArfusoRosario BaroneFrancesca BlasiRosario DonatoFrancesca CianiRosella CataldoFrancesca GaleffiRosita GabbianelliFrancesca GiampieriRosnah ShamsudinFrancesca MaccariRoss GrantFrancesca MeliniRossana MorabitoFrancesca RappariniRossana Rodríguez-CanulFrancesca Romana FuscoRossella PanareseFrancesca S. ForiniRossi LucianaFrancesca SciandraRoumiana P. StatevaFrancesca SilvagnoRoumiana StatevaFrancesca ViazziRoy MoncayoFrancesco BiancoRubén Francisco González-LaredoFrancesco CacciolaRudy OrtizFrancesco CaponioRuei-Nian LiFrancesco CiccareseRui LiuFrancesco De ChiaraRui M. Borges Dos SantosFrancesco Di GennaroRui OliveiraFrancesco Di LorenzoRui VitorinoFrancesco FazioRuilong ShengFrancesco FornaiRu-Lan HsiehFrancesco GaiRumyana SimeonovaFrancesco MeneguzzoRune WaagboFrancesco OrienteRunpu ChenFrancesco PallottiRupal I. MehtaFrancesco ScavelloRuslan MariychukFrancesco SessaRute CesárioFrancesco StiloRuth PrasslFrancesco VisioliRwik SenFrancisca RodriguesRyan MaillouxFrancisco B. FloresRyota HosomiFrancisco BerralRyszard AmarowiczFrancisco DasíRyszard CierpiszewskiFrancisco EspinosaSabbatini MaurizioFrancisco EstévezSabina Antonela AntoniuFrancisco García-CózarSabina GaliniakFrancisco García-MolinaSabina GalusFrancisco Gracia-CanovasSabina Lachowicz-WiśniewskaFrancisco Guillen-GrimaSabreen Ezzat FadlFrancisco Javier López RománSabrina DallavalleFrancisco Javier RomeroSabrina Donati ZeppaFrancisco José Gómez GarcíaSabrina TrudoFrancisco José González-MineroSachin GulatiFrancisco Lázaro-DiéguezSachio TakenoFrancisco LeonSaglara S. MandzhievaFrancisco LesSaida OrtolanoFrancisco PeixotoSaima ImranFrancisco SolanoSajal SenFranco CervellatiSaji UthamanFranco LucchiniSalma AbedelmalekFrank BraunSalvador E. Maestre PérezFrank H. QuinaSalvador MáñezFranko BurculSalvador MenaFrantišek DráfiSalvador PérezFrantisek HnilickaSalvatore BarrecaFranz RoedelSalvatore De RosaFranz ZehentmayrSalvatore FeoFritz SieberSamad RahimnejadFulvio MattiviSamantha S. SoldanFung-Fuh WongSameer J. MabjeeshFurong TianSami GhnimiG. Andres ContrerasSamooel JungGábor KocsySamson KheneGábor SomlyaiSamuel CaitoGabriel AullonSamuel E. AdunyahGabriel Goetten De LimaSamuel E. SchrinerGabriel HancuSamuel Fernández-ToméGabriel PlavanSamuel SilvestreGabriela HrckovaSanda AndreiGabriela Iulia DavidSanda WinGabriela PaunSandra GhelardoniGabriela RâpeanuSandra HillGabriela-Dumitrita StanciuSandra LeiszGabriele CarulloSandra MorenoGabriele Di MarcoSandra N. PintoGabriele Loris BeccaroSandra RebeloGabriele MorucciSandra SanchoGabriele MulthoffSandra SimõesGabriele SicilianoSang Gil LeeGabriella ChieffiSang Yeol LeeGabriella DobrowolnySang-Ha KimGabriella LeonarduzziSang-Han LeeGabriella PiroSang-Won LeeGabriella SarmaySang-Wook ParkGabriella TamasiSanjay GuptaGadi SchusterSankar JagadeeshanGaetano MarvertiSanta CirmiGaetano SantulliSantiago CuevasGagan FloraSantiago Diaz-OltraGaia RocchittaSantiago Garcia-GrandaGalina NikolovaSantiago P. AubourgGareth EatonSantosh UpadhyayGary GaoSapna SharmaGary MartySara Casado ZapicoGaurav SharmaSara DamianoGavin PinnigerSara De MartinGavino SannaSara FulignatiGederts IevinshSara GoldsteinGeert MayerSara Hurtado-BarrosoGeert PottersSara MarinelliGema NietoSara PerteghellaGengjun ChenSara PetrilloGennaro Mariano LenatoSarah M. ClarkGennaro Roberto AbbamondiSarah WalkerGeon A. KimSarantis GagosGeorg Daniel DuerrSarath VijayakumarGeorg F. WeberSarbani GhoshalGeorg VoelckerSaroj K. BasakGeorg WondrakSascha RohnGeorge AnifandisSathish Kumar NatarajanGeorge HsiaoSatoru KaseGeorge Mihai NitulescuSatoshi HagaGeorge ThurstonSatoshi MaruyamaGeorgina Galicia-RosasSatoshi MigitaGeorgios GalaziosSatoshi WaguriGeorgios PampalakisSaúl Gómez-ManzoGeraldine GueronSavino SpadaroGerard DijkstraSawsan ZaitoneGerardo Alvarez-RiveraSayantani ChowdhuryGerardo Fernández BarberoSe Jin ParkGerasimos P. SykiotisSean ChiaGergana MihailovaSebastian GranicaGergely FeherSebastian VogtGerhard PüschelSebastiano A. G. LavaGermán EbenspergerSébastien LeveneurGermano CastelliSebastien PomelGeta CaracSeema DangwalGiacinto BagettaSeema GuptaGiacomo PetrettoSeiichi MatsugoGiacomo ZacconeSe-Jae KimGiamberto CasiniSekhar P. M. ReddyGiampaolo MinettiSelestina GorgievaGiampaolo MorcianoSeok-Seong KangGiampiero CaiSeong Beom ChoGiampietro ViolaSeon-Yong JeongGian Carlo TenoreSerena LemboGian Luigi RussoSerena MirraGian Paolo LittarruSerge BottariGianandrea PasquinelliSerge BrandGiancarlo AldiniSergey DikalovGiandomenico CorradoSergey FedoreyevGiane Regina PaludoSergey KolomeichukGianfranco FontanaSergey KozlovGianfranco NataleSergio Pérez-BurilloGianfranco SantovitoSergio Rius-PérezGianluca CestraSerhat Sezai ÇiçekGianluca De MasiSerkos A. HaroutounianGianluca FulgenziSeth CoreyGianluca PaventiSeung Myung MoonGianluca RizzoSeung-Chun ParkGianluigi MazzoccoliSeung-Hyun RoGianna FerrettiSeung-Rock LeeGianni BattaconeSeverino Matias De AlencaraGideon GroganSeyed Khosrow TayebatiGilbert S. OmennShahid UmarGilles CurienShailendra SinghGilles LemaîtreShaimaa SelimGilles LemercierShalaka MulherkarGinés B. Martínez–HernándezShalini DograGinnae AhnShane ThomasGiordano PulaShanmuga S. MahalingamGiorgia CerqueniShaohua WangGiorgia OlivieroShao-Wen HungGiorgia PallafacchinaShaowu XueGiorgia SaraisShatadal GhoshGiorgio FacchettiSheldon RowanGiorgio RicciShenghuei LanGiosafatto Concetta Valeria LuciaSheng-Nan ChangGiovanna FiaShichen ShenGiovanna MottolaShigeki MatsubaraGiovanna TrainaShigeki SugawaraGiovanni CasiniShigenori KumazawaGiovanni Di BernardoShigeo OhtaGiovanni Francesco MarangiShihai ZhangGiovanni Mario PesShih-Min HsiaGiovanni MitaShiho OhnishiGiovanni PallioShihori TanabeGiovanni RibaudoShih-Yi HuangGiovanni RollaShin TakasawaGiovanni SignoreShingo KasamatsuGiovanni TarantinoShinichiro OchiGiovanni TossettaShining GuoGirish PattappaShinji TeramotoGisela BeutnerShinobu TakizawaGitana Maria AcetoShinya MatsuzakiGiulia AbateShiping BaiGiulia BianchiShiri LiGiulia DowgierShiro SuetsuguGiulia MatacchioneShohei MitaniGiulia SecciShoji TsujiGiuliana MuzioShojiro SawadaGiuliana SolinasShu XiaodongGiuliano RamadoriShubo JinGiulio CuroneShuen-Ei ChenGiulio Francesco RomitiShugo SuzukiGiulio VistoliShuichi MachidaGiuseppe AnnunziataShukho KimGiuseppe BiaginiShun-Fa YangGiuseppe De MarcoShu-Yao TsaiGiuseppe EspositoShyamsundar Pal ChinaGiuseppe LosurdoShyh-Jiun LiuGiuseppe ManninoShyi-Tien ChenGiuseppe MartelliShyue-Fang Battaglia-HsuGiuseppe MaulucciSi Ming ManGiuseppe Maurizio CampoSidonia MartínezGiuseppe MinerviniSieglinde ZelzerGiuseppe MurdacaSiemowit MuszyńskiGiuseppe PiccioneSihui MaGiuseppe PuglieseSilvana AlfeiGiuseppe SammarcoSilvana PedatellaGiuseppe SancesarioSilvano DragonieriGiuseppe ValacchiSilvano Junior SantiniGiuseppina AmadoroSílvia A. SousaGiuseppina De LucaSilvia BistiGiuseppina MandalariSilvia Di GiacomoGiuseppina MartellaSilvia FinnemannGladys KoSilvia Gómez-JiménezGladys Oluyemisi Latunde-DadaSilvia Graciela CorreaGlatz AttilaSilvia LandiGlenn LoboSilvia M. ArribasGloria E. O. BorgstahlSilvia MarracciGloria SalazarSilvia PalmaGobinath ShanmugamSilvia PampanaGodfrey GetzSilvia SelleriGoki SudaSilvia TampucciGonçalo J. JustinoSimon GalasGopi KolluruSimona DordevicGoran GajskiSimona FabroniGordana ĆetkovićSimona LattanziGordon LoweSimona Lucia BavaroGotthold GäbelSimona MartinottiGrace Y. LamSimona MirelGrace Y. SunSimona Mrakic-SpostaGraham R. McginnisSimona PergolizziGrazia Maria BorrelliSimona RapposelliGraziamaria CorbiSimona SabbatiniGrażyna BudrynSimona SagonaGrażyna LewandowiczSimona SerratìGrazyna NeunertSimona TerzoGrażyna NowickaSimona ZaamiGrażyna Odrowaz-SypniewskaSimone BattagliaGrażyna SygitowiczSimone BrogiGrazyna T. KochanSimone Di FrancoGrażyna ZgórkaSimone MessinaGregorio Barba-EspinŠimons ŠvirskisGregorio Martínez-SánchezSinisa VolarevicGrzegorz BartoszSiqi HuGrzegorz CholewinskiSlaven JurićGrzegorz ChrzanowskiSlaven ZjalićGrzegorz GrześkSławomir BorekGrzegorz LitwinienkoSławomir DreslerGrzegorz ŁysiakSławomir GonkowskiGrzegorz SchroederSławomir KulaGrzegorz TrybekSlawomir WybraniecGuadalupe Virginia Nevárez-MoorillónSławomir ZduńczykGuanbin SongSobhy El-SohaimyGuang-Chao ChenSofia LimaGuan-Jhong HuangSoichiro MurataGuanjun YangSokhna M. S. Yakhine-DiopGuarnier Flavia AlessandraSoliman KhatibGuglielmo DurantiSom DevGuiguang ChengSomiranjan GhoshGuillermo CásedasSon Dong-JuGuillermo Cristian Guadalupe Martínez ÁvilaSonani Ravi RaghavGuillermo GervasiniSonia BañuelosGuillermo López LluchSónia BatistaGuillermo PetzoldSonia BenitezGuillermo RodríguezSonia LaneriGuillermo Tellez-IsaiasSonia PiacenteGuillermo Zalba GoñiSonia ScarfìGunnar BoysenSonia SentellasGünter MüllerSonu Menachem Maimonides BhaskarGunther MarscheSoo Jin YangGuodong RaoSoochong KimGuohua GongSophie Thetiot-LaurentGuohua MaSoraia Katia Pereira CostaGuojun WuSorana IonescuGuoqing NiuSoraya L. VallesGurdeep MarwarhaSorin Marius AvramescuGurunathan JayaramanSowmya ViswanathanGuy HansSowmyalakshmi SubramanianGuy MechrezSoyeon LimGuy RostokerSpencer GibsonGuzel R. KudoyarovaSpyridon A. PetropoulosGwang-Woong GoSree V. ChintapalliGyörgy KeglevichSrinivas SriramulaHagir B. SulimanSsang-Goo ChoHaibo WangStacy Gelhaus WendellHaigh TonyStanislav N. NaryzhnyHaike AntelmannStanisław KaliszHaim BreitbartStanisław KwiatkowskiHaipeng XiaoStanisław WitkowskiHaitao YuanStanley ChanHajime KojimaStavros KalogiannisHajo HaaseStavros PlessasHåkan N. PärssonStefan LüthHåkan WesterbladStefan MihaicutaHakuto KageyamaStefan SajdakHalina JurkowskaStefan SchildknechtHan A. ParkStefan TimmHan MoshageStefan W. RyterHana BuchtováStefania CesaHana HansíkováStefania D’AngeloHana RauchováStefania FulleHan-Jia LinStefania GonfloniHan-Jun KimStefania MerighiHanna BaranowskaStefania MocciaHanna KmitaStefania SutHanna SzaeferStefano AlcaroHans G. DrexlerStefano CastellaniHanseob JeongStefano CecchiniHanspeter NaegeliStefano DocciniHany Hamdy ArabStefano FumagalliHao LiStefano GattiHao WangStefano LorenzettiHao-Jen HsuStefano LuinHao-Xun ChangStefano ManfrediniHaralambos StamatisStefano MenichettiHarald PlattaStefano PierettiHarald W. PlattaStefanos DailianisHarris PratsinisStela DimitrovaHatasu KobayashiStela JokićHaya FriedmanStella OrdoudiHayato MaedaStepan GambaryanHayato TerayamaStephan ImmenschuhHazel Monica PeraltaStephan KrähenbühlHeather WilkinsStéphanie AndradeHedvig FébelStephanie DuguezHee Kyoung JooStephanie HehlgansHee-Chul AhnStephanie PadillaHee-Shang YounStéphanie SimonciniHeinrich SauerStephen C. Kolwicz, Jr.Helen FogartyStephen CessnaHelena G. AzinheiraStephen CornishHelena L. A. VieiraStephen InbrajHelene A. FachimStephen J. PetersonHelga MoncayoSteven C. CookHemendra VekariaStrzemski MaciejHenning ArltStylianos ExarhopoulosHenry PleassSu Hyuk KoHerbert Ryan MariniSubhadeep ChakrabartiHeriberto Hernández-CocoletziSubhadip MukhopadhyayHerminia Gonzalez-NavarroSubhashree SubramanyamHermona SoreqSubramanyam DasariHernandes CarvalhoSudhahar VaradarajanHervé KovacicSudip BanerjeeHesham El–EnshasySuguru YamamotoHidehiko KikuchiSumino YanaseHideo KimuraSun MengtaoHideo SuzukiSun Young ParkHideyuki J. MajimaSung Ho MaengHideyuki KawauchiSung Hwan KiHideyuki MaedaSung Hyun KimHieronim JakubowskiSung Woo KimHilton DeethSungeun KimHinrich StaeckerSunghak KimHiran A. PragSung-Hoon KimHiroaki GotohSung-Jae LeeHirohito IshigakiSung-Jig LimHirokazu TamamuraSunil Kumar SahuHirokazu TsukaharaSurendra RajpurohitHiroki TeragawaSuresh RattanHiroki TeraokaSusana Anahi DandlenHiroki ToyodaSusana BernardinoHiromasa HasegawaSusana M. CardosoHiromi SakaiSusana OchoaHiroshi HaraSusana RochaHiroshi IchikawaSusanne BaldermannHiroshi KitagakiSusete PinteusHiroshi MiyamotoSusumu MuroyaHiroshi ShibuyaSusumu TanakaHiroshi TakemoriSuzana ŠegotaHiroyuki KataokaSuzana ŽunecHiroyuki MoriSven WürtzHiroyuki SakakibaraSvend LindenbergHiroyuki TsuchiyaSvetlana AidagulovaHiroyuki WakiguchiSvetlana KamzolovaHiroyuki YamadaSvetlana KlinovaHisashi MatsudaSvetlana P. ErmakovaHiu Chuen LokSvetlana SimovaHodur CeciliaSvetlana V. VeselovaHong MaSwapnil SonkusareHong Yong PehSwarup RoyHongbing FanSwati MoreHongdeng QiuSybille HasseHong-Duck KimSyed Bilal HussainHong-Jhang ChenSyed J. KhundmiriHongjie DaiSylvain BourgoinHonglian ShiSylvia PietriHongmei CaiSylvie RenaultHongmin WangSylwia MałgorzewiczHongming SuSze Wan Samantha ShanHongtao JinSzilvia BánvölgyiHongyi ZhouSzymon BocianHoon KimTadaaki SatouHorng-Huey KoTadeusz KowalskiHo-Shik KimTadeusz PawełczykHossam El-BeltagiTadeusz SarnaHossein ZareTadeusz StefaniakHrvoje LepedušTae Kon KimHsiu-Chen HuangTae Sung JungHsiu-Chuan ChouTae-Gyu LimHsiu-Mei ChiangTae-Hoo YiHsueh-Wei ChangTae-Kang KimHu HuangTae-Sun MinHua LiTaiki MiyazawaHua-Jun FengTai-Long PanHuakun ZhouTaisuke TomonagaHuaxin ShengTakaaki SenbonmatsuHubert ScharnaglTakahiro NemotoHuei-Jane LeeTakahiro Watanabe-NakayamaHugo Pequeno MonteiroTakahito MukaiHui ChenTakamitsu TsukaharaHui LiuTakao KimuraHui ZhaoTakashi MinegishiHui ZhuTakashi SatoHui-Chi HuangTakashi UeharaHui-Ching ChuangTakashi YazawaHui-Hua HsiaoTakayoshi WakagiHuilan YueTakayuki OkamotoHui-Yen ChuangTakehisa HanawaHung-Chi ChengTakeji Takamura-EnyaHung-Lung ChiangTakeshi AdachiHung-Pin TuTakeshi KitaiHungyi ChuangTakeshi SatoHunseung KangTakeshi UemuraHussein G. DaoodTakeshi YamamotoHye One KimTakujiro HommaHyemin LeeTakuma MatsubaraHyeyoung MinTakuya SakamotoHyon Sob HanTakuya SakuraiHyun Sook HongTamara Emmy LacourtHyung-Jun KimTamas KálaiHyung-Kyoon ChoiTamás LetohaHyun-Jae ShinTamás RöszerIain A. BrownleeTânia MartinsIain BrownleeTânia MonizIain D. CroallTanyalak ParimonIain Parry HargreavesTao FengIan L. MegsonTarja KokkolaIfat SherTatiana ArmeniIgnacio BadiolaTatiana ErazoIgnacio BejaranoTatiana GolubevaIgor BadoTatiana V. VygodinaIgor Piotr TurkiewiczTatiana WojciechowiczIgor SplichalTatsuhiko FurukawaI-Jung TsaiTatsuro MutohIkuo KashiwakuraTatyana PopovaIkuo NakanishiTatyana V. SavchenkoIl Soo MoonTed S. AcottIlaria BellezzaTeemu TeeriIlaria CampesiTeodor RusuIlaria PatiTeodora BasileIlaria PelusoTeodora Emilia ColdeaIlaria PianoTeodoro Coba De La PeñaIlaria ZanottiTeodoro PalomaresIlavenil SoundharrajanTeresa CarbonellIldikó HorváthTeresa CardosoIldiko RaczTeresa Inés Freire GardIldiko SzantoTeresa MencheriniIldikó UnkTeresa Sosa DíazIliana Medina-RamírezTeresa TrottaIlias GiannenasTeresa WitczakIliyan IvanovTero A. H. JårvinenIl-Man KimTerrence PivaIlya PinchukTerry D. HindsIlya ZavodnikTetsuro IshiiImmacolata CastellanoTetsuya KonishiImre LengyelThavaree ThilavechImre VassThea MagroneIn Koo HwangTheerthagiri JayaramanIna JasutieneTheodora PapamitsouIn-Beom KimTheodoros SklaviadisIn-Chul LeeThierry AussenacIndu DharThimios A. MitsiadisInes CastangiaThomas C. ThannickalInes DrenjančevićThomas CrenshawInes FasolinoThomas E. KuhlmanInés GarbayoThomas J. BachInês M. AraújoThomas O’ConnellInês PortugalThomas S. WeissInga KwiecienThunder JaliliIn-Hyun NamTiago A. FernandesInmaculada FernándezTiancai LiuInmaculada ParrillaTianhua NiuInna E. PopovaTianhui HuInna SolomonovTianyu LiInna ZamulinaTibor ErtlIoan NegulescuTiebing LiangIoana IonitaTien-Yuan WuIoanna NtaikouTieying YinIoannis A. TsakmakidisTilo LuebkenIoannis AnestopoulosTim FosterIoannis K. KarabagiasTim ReynoldsIoannis K. ToumpoulisTimoteo MarchiniIoannis KanakisTimothy E. O’TooleIoannis KourkoutasTimothy John TranbargerIoannis MorianosTina B. MckayIoannis RoussisTina MckayIoannis S. PaterasTina U. CohnertIoannis ZabetakisTing LiuIoannis-Dimosthenis AdamakisTiziana AlberioIonut LuchianTiziana BacchettiIosif MarincuTiziana CiarambinoIrena Brčić KaračonjiTiziana PietrangeloIrena IvshinaTiziano MarzoIrena NalepaTiziano VerriIrena SmagaTom HuxfordIrene DiniTom L. BroderickIrene RebeloTomás ÁlvaroIrene Russo KraussTomáš BrdičkaIreneusz KapustaTomas TakacIreneusz SowaTomas VenckunasIrfan RatherTomasz BlicharskiIrina G. GazaryanTomasz BrzozowskiIrina MilisavTomasz JelińskiIrina PopescuTomasz KostkaIrina Yu PetrushankoTomasz PoplawskiIrina ZarafuTomasz RusakIrinel Adriana BadeaTomasz SkrzypekIrineu BatistaTomasz ZarnowskiIris ScalaTomislav BosiljkovIrma ColomboTomislav MašekIrshad Ahmed HajamTomohiro MizobataIrwin Rose Alencar MenezesTomohiro SawaIsaac ZiporiTomoka Takatani-NakaseIsabel AndradeTomokazu KonishiIsabel Fort-GallifaTomomasa MatsuyamaIsabel Fuentes-CalvoTomotaka UgaiIsabel LarreTony MutukumiraIsabel Lastres-BeckerTorbjörn LedinIsabel María MorenoToru YoshitomiIsabel MarquesToshiaki AraIsabel Odriozola-SerranoToshiaki NakanoIsabel RodriguezToshihiro KitaIsabel SeiquerToshikazu SuzukiIsabel Torres-CuevasToshiro AraiIsabel VeladaToshiya NakamuraIsabella RussoToyoaki OhbuchiIsabelle DonnayTriantafyllos DidangelosI-Shiang TzengTrias ThireouIsmael León-RiveraTrinidad De MiguelIstván A. KrizbaiTristan DarlandIstván EgerszegiTrong TranIstván KissTse-Min LeeIstvan NagyTsung-Han LeeIstván PappTsutomu HatanoIstvan ZupkoTsutomu ShimuraI-Ta LeeTsvetelina BatsalovaItamar RazTuba EsatbeyogluIuliana SpiridonTung-Hsun HsiehIva ChianellaTuo ShaoIva PotočnjakTusty-Juan HsiehIvaldo ItabaianaTyson GraberIvan Cruz-ChamorroTze-Kiong ErIvan DimauroTzi Bun NgIvan GoutTzong-Shyuan LeeIvan KreftTzuen-Rong TzengIvan KushkevychTzung-Yan LeeIvan M. SavicUdaiyappan JanakiramanIvan SalmerónUdo JeschkeIvana CacciatoreUdo SeedorfIvana CavoskiUjendra KumarIvana KmetičUlf BickmeyerIvana NovakovicUlf SchottIvana Savic GajicUlrich GergsIvana Tlak GajgerUlrich WalterIvanka TsakovskaUlrika Marie NordströmIveta BernátováUlrike BaschantIveta PlacháUlrike BrüningIvna ŠtolfaUmberto MoscatoIvna Štolfa ČamagajevacUrsula Gundert-RemyIvo HavlikUrszula SzymanowskaIvonne Bazwinsky-WutschkeVadim ByvaltsevIwona CiereszkoVadim KlimontovIwona KonopkaVal C. SheffieldIwona ŚcibiszValdir Florencio Da Veiga JuniorIwona Wojtasik-KalinowskaValentin ReutovIwona ZwolakValentina CecariniIyyakkannu SivanesanValentina MeliniIzabela FeckaValentina PavicIzabela Nawrot-HadzikValentina PozziJ. Cameron MillarValentina RimondiJacco BriedéValentina VelleccoJacek AntonkiewiczValentyn OksenychJacek E. NyczValeria CarinaJacek ZelionkaValeria ContiJacinto Efrén Ramírez-BribiescaValeria MarigoJackob MoskovitzValeria MericoJacob RaberValeria PasciuJacqueline HegerValeria RighiJacqueline M. BentelValeria SogosJacques ZimmerValerian DormoyJadwiga Jodynis-LiebertValerie AmargerJadwiga ManiewskaValérie Schini-KerthJadwiga TurłoValerija DunkićJae Seung KangValery BochkovJae-Hyung ParkValter TravagliJaeyoon KimVan Khanh NguyenJae-Young JeVance G. NielsenJagna Chmielowska-BąkVanessa PorriniJaime EscalanteVanessa VieiraJakub CiazelaVasanth KumarJakub Patryk PiwowarskiVasco BrancoJakyeom SeoVasil SimeonovJalil Ghassemi NejadVasileios G. PapatsirosJamal BouitbirVasileios PapaliagkasJames A. MarrsVasileios ZiogasJames B. HurleyVasilica BarbuJames CobleyVasilii Vitalievich PtushenkoJames J. DriscollVassilis AthanasiadisJames J. MccormickVassilis L. TzounakasJames MacleanVasso OreopoulouJames ScholeyVassya BankovaJan BocianowskiVasudevan ManiJan Darius UnterlauftVed Prakash VermaJan GramsbergenVehary SakanyanJan Jacek KaczorVenediktova NatalyaJan JežekVenera CardileJan LehotskyVenkateshwar MadkaJan Lj. MiljkovićVera BonilhaJan PithaVera IsaacJana PickovaVera Luiza CapelozziJana RadosinskaVera MuccilliJana ŠtefánikováVera PanzarellaJanez MravljakVerena SpieglerJanina GospodarekVerena TischlerJanja TrcekVerónica BastosJanko MrkunVeronica DoderoJános G. FilepVeronika HuntosovaJanusz BłaszczykVeronika KanaJara Pérez-JiménezVéronique ImbertJarogniew ŁuszczkiVicente Martí-CentellesJaromír MyslivečekVicente NotarioJaroslav TóthVictor B. LoschenovJarosław BystrońVictor BanerjeeJarosław J. SakVictor E. LaubachJarosław L. PrzybyłVictor I. BandJarosław PrzybyłVíctor M. RiveraJarosław WidelskiVictor PulgarJaroslawa RutkowskaVictor TapiasJasenka Gajdoš KljusurićVictor VictorJasminka TalapkoVictoria Ramírez GonzálezJasna Čanadanović BrunetVictoria RoshchinaJasna Prlić KardumVictoriano MarceloJason CollierVida RezarJason ParsonsVida ŠimatJason T. C. TzenVidula VachharajaniJason Thomas DuskeyVijay HegdeJaume AmengualVikas YadavJaved AhmadViktor DrganJavier Caballero-VillarrasoViktória HornokJavier InserteViktoria JeneyJavier MateoVilceu BordignonJavier Murciano-CallesVilma KaškonienėJavier SaurinaVinayak KhodadeJaw-Yuan WangVincent JaquetJaya GnanaprakasamVincent LaizéJayanta Kumar PatraVincent SolJayasri DassharmaVincenza GianfrediJean Daniel CoïssonVincenzo BrancaleoneJean Michel MaixentVincenzo De FilippisJean RegalVincenzo De LeoJeanette A. M. MaierVincenzo MicaleJean-Luc MorelVincenzo MollaceJean-Marc ZinggVincenzo NicolettiJean-Marie RuysschaertVincenzo ParrinoJedlińska AleksandraVincenzo QuagliarielloJee-Young ImmVincenzo SummaJeff VictoroffVincenzo TufarelliJeffrey Bryant TraversVioleta PeevaJeffrey DowningVioletta MacioszekJeffrey WhiteVipin Chandra KaliaJeffrey WigleVirgínia Cruz FernandesJegan IyyathuraiVirginie Monnet-CortiJelena DinicVita De StefanoJelena KnezevicVitaly A. SorokinJelena KrsticVittorio CalabreseJelena ViderVittorio LocatelliJelli VenkateshViviana GrecoJennifer C. HockingViviana VergaroJennifer GambleViviane VerlhacJennifer GubitosaVlad PădureanuJennifer MytychVlad SerbuleaJennifer PeelerVladimir Anatolievich ChistyakovJennifer SneddonVladimir DvoretskyJens FielitzVladimir GureevJeny Rachid Cursino-SantosVladimir K. IvanovJeong DongtakVladimir KefalovJeong-Hun KangVladimir MrsaJeong-Hwan KimVladimir NikolenkoJeovanis GilVladimir ReukovJer-Ming ChangVladimir SerezhenkovJerneja JakopicVladimir V. BezuglovJérôme PiquereauVladimir VladimirovJérôme RoyVladislava GusarJérôme ZodiakisVlasios GoulasJer-Yiing HoungVlastimil KuldaJerzy ChudekVlatka GvozdićJerzy JuśkiewiczVolker BehrendsJesús Fernando Ayala-ZavalaVolker BöhmJesús M. MercadoWajid ZamanJesús M. MíguezWaleed MareiJesús PalomeroWallace K. MacnaughtonJesús TejeroWanda F. ReynoldsJesus V. Jorrin-NovoWanda MączkaJe-Wen LiouWan-Wan LinJezabel Rodriguez-BlancoWayne ThomasJiakui LiWeber Andre AlbertoJian LiWei ZhongJian ZhangWei ZhuJiangang ShenWei-Biao LiaoJianmei YuWeien YuanJianming KangWeilin JinJiann-Chu ChenWei-Lun HungJiann-Jou YangWei-Min ChenJianqiang XuWeimin HuangJia-Yaw ChangWei-Wen KuoJiayu ZhuWeixing ZhaoJichang WangWellison Jarles Da Silva DinizJie FuWen XieJihane Homman-LudiyeWen-Bin WuJih-Huah WuWen-Chi HouJiiang-Huei JengWen-Chieh SungJillian MadineWen-Chien LuJimin GuoWen-Chin HuangJin Ah ChoWendy BollagJin LeeWen-Hsing ChengJinan LiWenji PiaoJin-Chul KimWenjun ZhangJing XingWenqun LiJingyan HanWen-Tsong HsiehJinhae KimWenxin FuJinhua ChengWesley KephartJin-Kyung KimWesley SwingleyJinning LiuWiesław BielasJinwei ZhangWiesław PrzybylskiJinxiang XiWiesława JarmuszkiewiczJin-Yong LeeWilhelm BlochJiri KassaWilhelm J. BaaderJiri PopelarWilhelm MosgöllerJiro HaradaWilliam DuranteJiuann-Huey Ivy LinWilliam G. MayhanJiunn- Liang KoWilliam K. F. TseJoan LiWilliam L. StoneJoana CarvalhoWilliam Mark ErwinJoana RelatWilliam N. SetzerJo-Ann C. LeongWilliam R. ReedJoanna Bartkowiak-WieczorekWilliam SelfJoanna BartosinskaWilliam T. Gunning IIIJoanna BrysWillibald WonischJoanna GajewskaWindson Isidoro Haminiuk CharlesJoanna Gdula-ArgasińskaWinfried SteilingJoanna GiebultowiczWioletta MędrzyckaJoanna Gromadzka-OstrowskaWitold KędzierskiJoanna KoreckaWitold KorytowskiJoanna LeszczyńskaWladyslaw KordanJoanna LiszkowskaWładysław LasońJoanna Perła-KajánWojciech KalasJoanna RzemieniecWojciech KochJoanna SuliburskaWojciech ŁuczajJoanna TimminsWojciech PiekoszewskiJoão Agostinho Machado-NetoWolfgang MaretJoao F. ManoWolfgang SattlerJoão Guilherme CostaWolfram KunzJoão Henrique Ghilardi LagoWonhyo SeoJoão Paulo CapelaWon-Kyu LeeJoão Ramalho-SantosWooyeong ParkJoão VicenteWulf PalinskiJoaquim CarrerasWupeng LiaoJoaquín María CamposXavier GuillonneauJochen D. SchipkeXavier XifróJochen SchneiderXi ZhouJoe H. BanoubXian-Cheng JiangJoe VinsonXiang GaoJoël PincemailXiang LiJoel S. GreenbergerXiangnan LiJoel T. MagueXiangping ChuJoellyn M. McmillanXiangyang LiJoerg DurnerXiao Fei LiJóhannes ReynissonXiao-Bin ZhangJohannes Von LintigXiaobo MaoJohn A. TayekXiaochuan PanJohn AdamovicsXiaohan TangJohn Bertram VincentXiaojun DuJohn CarragherXiaomei HeJohn D. HorowitzXiaoqiu ZhouJohn E. PiletzXiaoye YangJohn EllulXiaoyu SongJohn H. MillerXin ChenJohn HonekXin DuJohn HullemanXin LiJohn J. LapresXin LuoJohn J. SkokoXin WangJohn Kelly SmithXin ZhangJohn O. OnukwuforXing WangJohn P. HulmeXing ZhangJohn W. YuenXinli LiJohn WuXiqian JiangJolán CsiszárXiumin FuJolanta JaworekXiuxiu SunJolita RadušienėXuewu DuanJonathan S. TamXugan WuJones Ng’AmbiYahya SohrabiJong-Eun KimYajing WangJong-Hyun JungYan ZhangJongki ChoYana SandlersJong-Tae ParkYanan WangJong-Won KimYang ZhaoJoohyung LeeYang-Ju SonJorddy Neves CruzYan-Hsiung WangJörg HänzeYanjun GuoJörg PetersYan-Ming XuJorge CervantesYannis KotzamanisJorge FeitoYanshu ShiJorge GonçalvesYashomati M. PatelJorge Luis García-AlcarazYasser KandilJorge Moreno-FernandezYasuaki TakagiJorge PereiraYasuhiro KosugeJosé A. BritoYasuka MatsunagaJosé Agustín Tapia HernándezYasuko MoriyamaJose Angel FontenlaYasumasa OkazakiJose Angel Pérez-ÁlvarezYasumichi InoueJosé Antonio BeltránYasuo IdoJose Antonio De Paz FernándezYasuo WatanabeJosé Antonio PérezYasushi HasegawaJose CanasYasuyuki NagasawaJose Contreras-CalderónYaw L. SiowJosé De Jesús Ornelas-PazYehong ZhuoJosé Enrique Torres VaamondeYelena V. ParfyonovaJose Felix Moruno ManchonYeonghsiang ChengJosé G. Fernadez-BolanosYeong-Min YooJosé Luis Guil-GuerreroYichun TsaiJose Luis Marco-ContellesYi-Jang LeeJosé M. MatésYimei JiaJose M. MuletYing-Hsien KaoJosé M. Pérez-OrtizYingtao LiuJosé Manuel UgaldeYiqun DengJosé Marco-ContellesYiqun MoJosé María Moreno-NavarreteYi-Sheng ChengJosé María Palacios-SantanderYi-Ting WangJosé Martín-NietoYi-Yin DoJosé Miguel P. Ferreira De OliveiraYlenia SpissuJosé Néstor CaamañoYo SasakiJosé Pedraza ChaverriYoana KiselovaJosé PinelaYoana Rabanal RuizJosé Rodriguez-RodriguezYoav SharoniJose Sanchez-AlcazarYogesh VikalJosé Sousa CâmaraYohei IkezumiJose TudelaYoko HirataJosef IllekYoko Kimata-ArigaJosef VelíšekYoko YagishitaJoseph A. DiverdiYong AiJoseph A. M. J. L. JanssenYong Chool BooJoseph A. WestrichYong GeJoseph CullenYong JinJoshua D. KleinYong TangJoshua KleinYongjun WeiJosiana A. VazYong-Pil CheonJosip DelmisYong-Suk KimJosipa VlainićYorito HattoriJosko BozicYosef ShamayJosy AugustineYoshiaki YuraJoszef DudasYoshifumi SaishoJózef DrabowiczYoshiharu FujiiJózsef DeliYoshihiro FukumotoJuan A. PargaYoshika SekineJuan Antonio Giménez-BastidaYoshimi NiwanoJuan AyalaYoshinori UtsumiJuan José MorenoYoshitaka NakayamaJuan José Ramos-RodríguezYoshiteru MorioJuan Luis Gómez PinchettiYou-Cheng ShenJuan Manuel Mejía-AranguréYouji WangJuan NavarroYoung Dal JangJuan Pablo MuñozYoung Min ChoiJuan PeragónYoung-Eun ChoJuan PouYoung-Ok SonJuan R. De Los ToyosYoung-Woo KimJuan R. Muñoz-CastañedaYousif SubhiJuan SanabriaYoussef HibaouiJude OkolieYoussef RouphaelJudit KrischYoussra Al-HilalyJudit OláhYousuf MohammedJudyta Cielecka-PiontekYousuke TaokaJuei-Tang ChengYu Chan ChangJuergen ArnholdYuan NieJuergen SchrezenmeirYuan Shiun ChangJui-Yang LaiYuan-Yen ChangJukrapun KomaikulYuanyuan XuJulia BrenmoehlYub Raj NeupaneJuliann G. KiangYuchao ChangJulio César Almanza-PérezYuchun DuJulio OchoaYueh ChoJulita KulbackaYuesheng DongJulius BogomolovasYuh BabaJun FangYu-Hsiang KuanJun ItamiYu-Hsuan LeeJun LuYu-Hung LinJun MaYuichi TsuchiyaJun Sik LeeYuichiro J. SuzukiJun ZhouYuichiro YamashiroJun-Hong LiuYuji ShimizuJun-Ichi KadokawaYuki EnokiJunichi ShodaYukinori YabutaJunling GuoYu-Kuo ChenJunping YuYulia BaburinaJunsei MimuraYu-Ling LinJunseo OhYuliya I. RaginoJunsoo LeeYulong TangJunyan JinYun HuangJunyang JungYun ShiJu-Ock NamYun ShimJuozas LazutkaYun-Chin ChungJuraj MajtanYunfeng ZhaoJuraj PiestanskyYung-Chia ChenJurandir F. ComarYung-Hsiang TsaiJürgen SchlegelYunqian DaiJustus G. GarwegYun-Wen ZhengJustyna BohaczYury LadilovJustyna LiberaYuseok MoonJustyna SikoraYusheng LiangJustyna SzumiłoYusra Habib KhanJwu-Lai YehYusuke HirataK. J. Senthil KumarYusuke OshimaKa YangYusuke TsukataniKai CaiYuta HatoriKai-Lee WangYutong ZhaoKaja KasarełłoYu-Tsai LinKajetan DabrowaYuwares MalilaKali MakedouYuxi FengKalina AlipievaYuya KumagaiKamel HessiniYu-Yi ChanKamil KaminskiYuyun LuKamil KarolczakYves HsiehKamil KrawczynskiYves-Jacques SchneiderKamlesh BhopaleŻaneta Kimber-TrojnarKamran AkbarZapryana R. DenkovaKamyar ZahediZbigniew MiszalskiKanae MureZdenék KejíkKankuri EskoZdeněk KejíkKapka MitevaZdeněk MoravecKarel AllegaertZeljan MalesKarel D. KlikaŽeljana FredotovićKarel KroftaŽeljko AndabakaKaren LibyZengqian ShiKarin HufnaglZenon PidsudkoKarin Zitterl-EglseerZenon WęglarzKarl StraubZhang MinKarmela BarisicZhanyong GuoKarmveer SinghZhao WangKarol OndriasZhaohui ChuKarola Vorauer-UhlZhe JinKarolina A. Wojtunik-KuleszaZhengan ZhangKarolina M. StepienZhengwei HuangKarolina Szewczyk-GolecZhengyu JinKaroly GulyaZheqi LiKarsten GroteZhi YangKatarína BauerováZhibin LiangKatarína ValachováZhicheng (Carl) LinKatarina VukojevicZhigang LiKatarzyna BączekZhihai LiKatarzyna JandaZhihui ChenKatarzyna KaczyńskaZhihui FengKatarzyna KlimekZhiqi CongKatarzyna KorybalskaZhizhi DuKatarzyna KoziakZhong LiKatarzyna KrukiewiczZhongbing LuKatarzyna NowomiejskaZhongjian ChengKatarzyna PobiegaZhongqi HeKatarzyna RatuszZhongwei HuangKatarzyna RoszekZhoufei WangKatarzyna RybakZhuoxun WuKatarzyna SobierajskaZhuqiu JinKatarzyna Sułkowska-ZiajaZia Shariat-MadarKatarzyna Sykłowska-BaranekZijuan LiuKatarzyna WalczakZipora TietelKatarzyna WińskaZishan ZhangKatarzyna Winsz-SzczotkaŽivoslav TešićKatarzyna WozniakZoltan M. VargaKatarzyna ZawadaZongjie WangKatarzyna ZorenaZongkang ZhangKateřina KaňkováZoran TodorovićKaterina RoubalovaZorita DiaconeasaKaterina VaporidiZoya OkunKatheleen J. GardinerZsuzsanna MiklósKathleen ReinZuhaibfayaz BhatKathrine BakZul’Fiya Sagdullaevna KhodzhaevaKatia ManganoZulfiqar Ahmad RehanKatia PetroniZuzanna RzepkaKatrin PanzittZvonko RumboldtKatrin SchröderZygmunt Warzecha


